# Protein disulfide isomerase is essential for spermatogenesis in mice

**DOI:** 10.1172/jci.insight.177743

**Published:** 2024-06-24

**Authors:** Yaqiong Zhang, Aizhen Yang, Zhenzhen Zhao, Fengwu Chen, Xiaofeng Yan, Yue Han, Depei Wu, Yi Wu

**Affiliations:** 1 National Clinical Research Center for Hematologic Diseases, Cyrus Tang Medical Institute, Collaborative Innovation Center of Hematology, State Key Laboratory of Radiation Medicine and Prevention, Soochow University, Suzhou, China.; 2 National Clinical Research Center for Hematologic Diseases, Jiangsu Institute of Hematology, Institute of Blood and Marrow Transplantation, Collaborative Innovation Center of Hematology, First Affiliated Hospital of Soochow University, Suzhou, China.

**Keywords:** Cell biology, Reproductive biology, Protein misfolding, Reproductive biochemistry

## Abstract

Spermatogenesis requires precise posttranslational control in the endoplasmic reticulum (ER), but the mechanism remains largely unknown. The protein disulfide isomerase (PDI) family is a group of thiol oxidoreductases responsible for catalyzing the disulfide bond formation of nascent proteins. In this study, we generated 14 strains of KO mice lacking the PDI family enzymes and found that only PDI deficiency caused spermatogenesis defects. Both inducible whole-body PDI-KO (*UBC-Cre/Pdi^fl/fl^*) mice and premeiotic PDI-KO (*Stra8-Cre/Pdi^fl/fl^*) mice experienced a significant decrease in germ cells, testicular atrophy, oligospermia, and complete male infertility. *Stra8-Cre/Pdi^fl/fl^* spermatocytes had significantly upregulated ER stress–related proteins (GRP78 and XBP1) and apoptosis-related proteins (Cleaved caspase-3 and BAX), together with cell apoptosis. PDI deletion led to delayed DNA double-strand break repair and improper crossover at the pachytene spermatocytes. Quantitative mass spectrometry indicated that PDI deficiency downregulated vital proteins in spermatogenesis such as HSPA4L, SHCBP1L, and DDX4, consistent with the proteins’ physical association with PDI in normal testes tissue. Furthermore, PDI served as a thiol oxidase for disulfide bond formation of SHCBP1L. Thus, PDI plays an essential role in protein quality control for spermatogenesis in mice.

## Abstract 2

Protein disulfide isomerase deficiency causes oligospermia and upregulates GRp78, XBP1, cleaved caspase 3, and BAX in spermatocytes.

## Graphical abstract

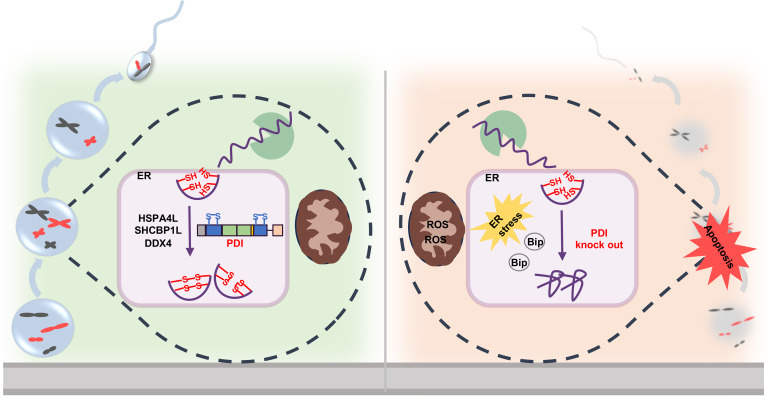

## Introduction

Spermatogenesis is a highly complex process by which male germline stem cells proliferate, migrate, and differentiate to produce mature haploid spermatozoa (1). There are 3 typical stages: mitosis of spermatogonia, meiosis of spermatocytes, and spermiogenesis of spermatids (2). These processes are subject to complex and precise control at transcription and translation levels, directing the normal expression of specific genes at different stages. Abnormalities at any step may result in meiosis arrest, spermatogenesis failure, and ultimately defective reproductive development. Using genetically modified mouse models, numerous molecules have been identified — such as HSPA4L, SHCBP1L, and DDX4 — that play crucial roles in spermatogenesis (3–5). However, how the synthesis of these proteins is regulated remains unknown.

The endoplasmic reticulum (ER) is the organelle responsible for protein synthesis and maturation. The protein disulfide isomerase (PDI) family is a group of multifunctional ER enzymes that catalyze the formation of disulfide bonds and is responsible for the quality control of nascent proteins to maintain proteostasis (6, 7). Given the rapid proliferation and differentiation of spermatogonia into spermatocytes, the ER-related mechanism is tightly involved in proper and efficient protein synthesis for spermatogenesis. Recent structural and functional studies have suggested that PDI family enzymes are involved in spermatogenesis. PDIA3 (also known as ERp57) was found in the cytoplasm of human testicular spermatogenic cells and the acrosome and flagella of human sperm (8). Another study confirmed that PDIA3 protein exists in cells at all stages of rat spermatogenesis as well as in Leydig cells, and *Pdia3* mRNA is similarly transcribed in cells at almost all stages of rat spermatogenesis (9). Moreover, PDIA3 was expressed on the sperm surface and was a component of sperm-egg zona pellucida binding complexes from the cell membrane of capacitive sperm. Functional blockade of PDIA3 with antibodies or inhibitors reduced the surface mercaptan content and zona pellucida binding ability of human sperm (10). In addition, PDIA6 (also known as ERp5), another member of the PDI family, was associated with heat shock protein A2 (HSPA2) in membrane raft microdomains surrounding the acrosome of the sperm head, suggesting that PDIA6 might contribute to fertilization cascade (11). More interestingly, the deletion of PDIA7 (also known as PDILT) in the testis caused infertility in mice, due to the decreased expression of a disintegrin and metalloproteinase 3 (ADAM3), which is a sperm membrane protein that is essential for the migration of sperm from the uterus to the oviduct; the role of PDIA7 is associated with disulfide bond formation of ADAM3 for its expression on sperm surface (12). A more recent study indicated that, in the sperm of a Chinese mitten crab (*Eriocheir sinensis*), PDI was expressed in the cytoplasm, cell membrane, and extracellular matrix in the spermatogonia, spermatocytes, and stage I and stage II spermatids and was mainly localized in the nuclei of stage III spermatids and sperm (13). Taken together, these previous observations suggest that the PDI family plays a key role in spermatogenesis; however, their exact functions have never been carefully characterized using genetically modified models. As a consequence, the mechanism of proteostasis necessary for spermatogenesis remains unknown.

In this study, we generated 14 strains of PDI family member gene–KO mice to identify their role in spermatogenesis in mice. We found that the deletion of PDI in premeiotic germ cells disrupted homologous recombination in the meiosis of spermatocytes causing complete male infertility, although the gene deletion of other members did not show an obvious phenotype. Our data provide the first genetic evidence to our knowledge demonstrating that PDI is selectively essential for spermatogenesis.

## Results

### PDI is expressed in testes and its deficiency selectively causes significant germ cell reduction.

**Figure 1:**
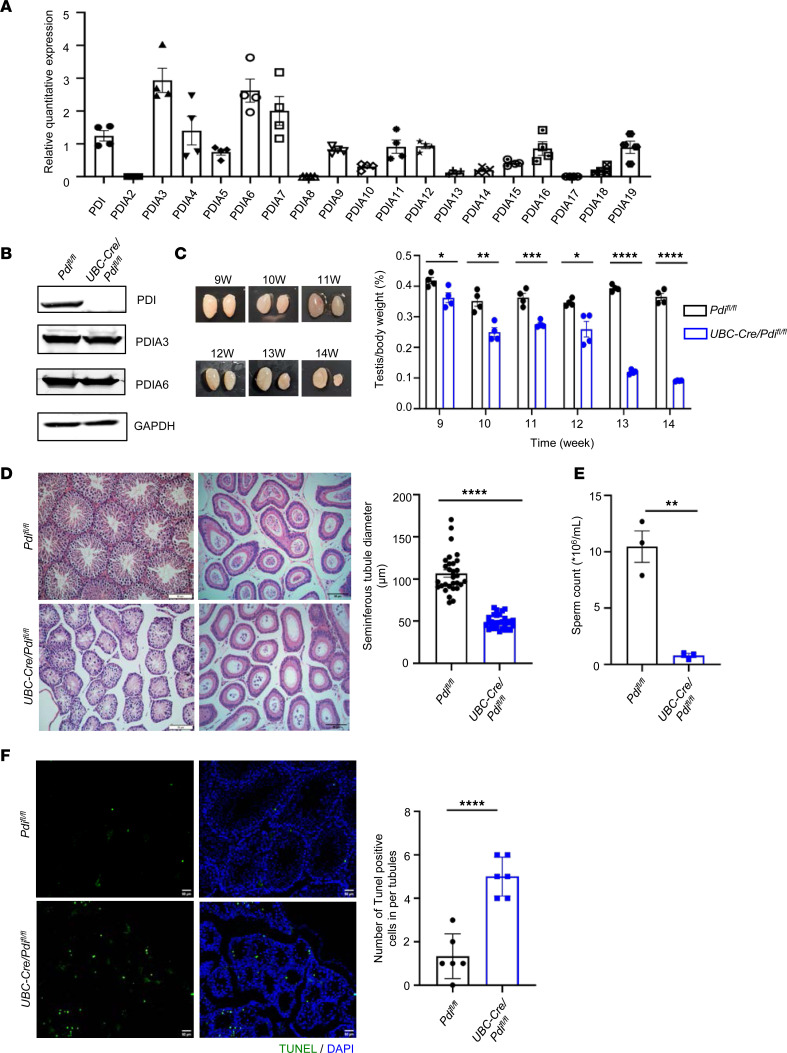
PDI is expressed in testes and its deficiency selectively causes significant germ cell reduction. (**A**) qPCR analysis of PDIs mRNA levels in adult mouse testes, normalized to GAPDH (mean ± SEM, *n* = 4, Student’s *t* test). (**B**) Western blotting analysis of testes proteins in control and *UBC-Cre/Pdi^fl/fl^* mice at 13 weeks old. GAPDH as an internal loading control. (**C**) Images of testes from control and *UBC-Cre/Pdi^fl/fl^* mice at 9–14 weeks old. Quantification of testes weight/body-weight ratios (mean ± SEM, *n* = 4, *P < 0.05, **P < 0.01, ***P < 0.001, ****P < 0.0001, Student’s *t* test). (**D**) H&E-stained testes and epididymis of control and *UBC-Cre/Pdi^fl/fl^* mice at 13 weeks old. Statistical analysis of seminiferous tubule diameter (mean ± SEM, *n* = 3, ****P < 0.0001, Student’s *t* test). Scale bars: 50 μm. (**E**) Sperm counts from adult control and *UBC-Cre/Pdi^fl/fl^* mice (mean ± SEM, *n* = 3, **P < 0.01, Student’s *t* test). (**F**) TUNEL assay of testes sections from control and *UBC-Cre/Pdi^fl/fl^* mice at 13 weeks old (mean ± SEM, *n* = 3, ****P < 0.0001, Student’s *t* test). Scale bar: 50 μm.

First, we measured the mRNA levels of the PDI family in adult male mouse testes using quantitative PCR (qPCR) and found high expression of *Pdi*, *Pdia3*, *Pdia6*, and *Pdia7* at mRNA levels (Figure 1A). In the previous studies, we introduced the KO mouse strains lacking PDIA3 (also known as ERp57) (14), PDIA4 (also known as ERp72) (15), and PDIA11 (TMX1) (16). We also generated KO strains deficient of other 11 members of this family, including PDIA1 (PDI), PDIA2 (PDIp), PDIA5 (PDIr), PDIA6 (ERp5), PDIA8 (ERp27), PDIA9 (ERp29), PDIA13 (TMX3), PDIA14 (TMX4), PDIA15 (ERp46), PDIA16 (ERp18), and PDIA19 (ERdj5). We found that the mice lacking only PDI had atrophied testes, but other KO mice did not. *Pdia2^–/–^*, *Pdia4^–/–^*, *Pdia5^–/–^*, *Pdia8^–/–^*, *Pdia9^–/–^*, *Pdia11^–/–^*, *Pdia13^–/–^*, *Pdia14^–/–^*, *Pdia15^–/–^*, *CAG-Cre/Pdia16^fl/fl^*, and *Pdia19^–/–^* mice had normal testes morphology and regular spermatogenic tubules, and they produced normal offspring (Supplemental Figure 1; supplemental material available online with this article; https://doi.org/10.1172/jci.insight.177743DS1 and data not shown). Because we knew in our previous study that the deletion of PDI in the whole body is embryonically lethal (17), the tamoxifen-driven conditional *UBC-Cre/Pdi^fl/fl^* mouse model was used (18, 19). The expression of PDI was absent in testes of *UBC-Cre/Pdi^fl/fl^* mice, although the expressions of PDIA3 and PDIA6, which share the 2 similar CGHC activation motifs, and other members were comparable with those of their littermate control mice (Figure 1B and data not shown). We found that, in male *UBC-Cre/Pdi^fl/fl^* mice, the testes gradually atrophied and decreased in both volume and mass (Figure 1C). Moreover, these PDI-deficient mice exhibited vacuoles in spermatogenic tubules, multinucleate bodies, absence of spermatocytes at all levels, and no round spermatozoa or elongated spermatozoa (Figure 1D). The sperm count was markedly reduced in the epididymis of *UBC-Cre/Pdi^fl/fl^* mice, compared with control *Pdi^fl/fl^* mice (0.79 × 10^6^ ± 0.18 × 10^6^/mL vs. 10.47 × 10^6^ ± 1.39 × 10^6^/mL; Figure 1E). TUNEL staining of PDI-KO testicular sections showed a triple increase in apoptotic cells over the controls (Figure 1F), suggesting that the PDI deficiency induces apoptosis of germ cells, causing the defect in spermatogenesis.

### Premeiotic germ cells PDI deficiency results in male infertility.

**Figure 2:**
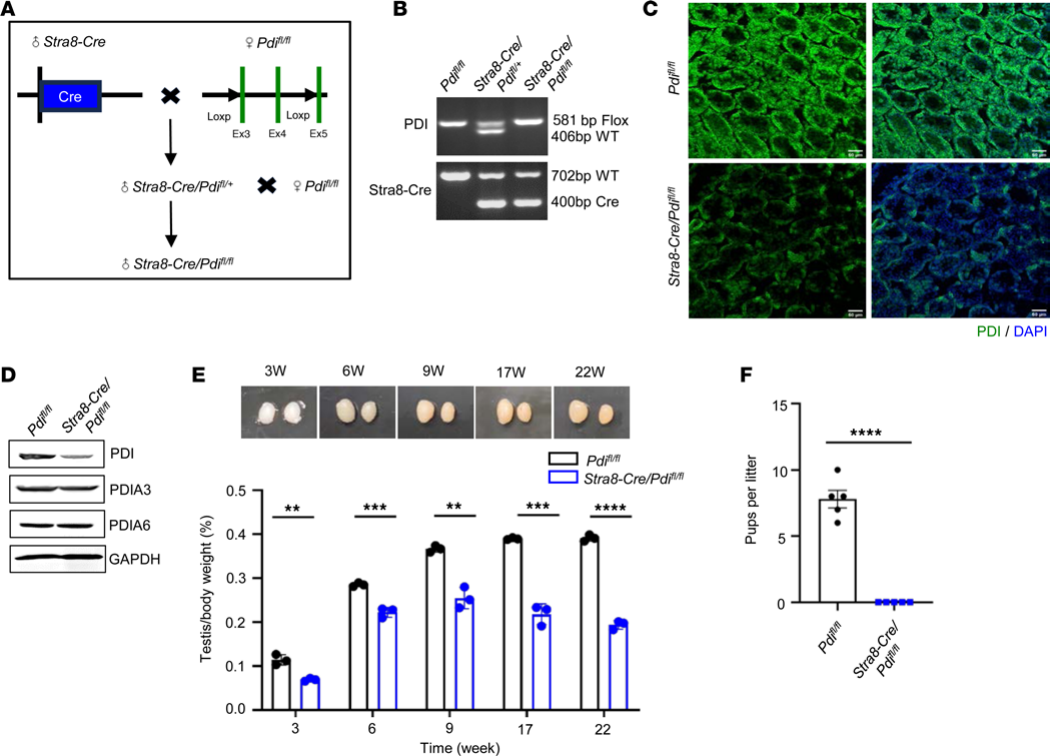
Premeiotic PDI deficiency results in male infertility. (**A**) The schematic strategy to generate spermatocyte-specific PDI KO (*Stra8-Cre/Pdi^fl/fl^* mice) by crossing *Pdi^fl/fl^* mice with *Stra8-Cre* mice. (**B**) Genotyping of *Pdi^fl/fl^*, *Stra8-Cre/Pdi^fl/+^*, and *Stra8-Cre/Pdi^fl/fl^* mice. (**C**) Immunofluorescence staining of PDI in control and *Stra8-Cre/Pdi^fl/fl^* mice at 2 weeks old. Scale bar: 50 μm. (**D**) Western blotting analysis of testes proteins in adult control and *Stra8-Cre/Pdi^fl/fl^* mice. GAPDH as an internal loading control. (**E**) Images of testes from control and *Stra8-Cre/Pdi^fl/fl^* mice at 3, 6, 9, 17, and 22 weeks old. Quantification of testes weight/body weight ratios (mean ± SEM, *n* = 3, **P < 0.01, ***P < 0.001, ****P < 0.0001, Student’s *t* test). (**F**) Fertility test of control *Pdi^fl/fl^* mice and *Stra8-Cre/Pdi^fl/fl^* male mice. In each group, five 3-month-old male mice were individually mated with 2 WT female mice for 3 continuous months (mean ± SEM, *n* = 5, ****P < 0.0001, Student’s *t* test).

To further confirm the role of PDI family members in spermatogenesis, *Pdi^fl/fl^* mice with *Stra8-Cre (Stra8-Cre/Pdi^fl/fl^*) (Figure 2, A and B) and mice lacking spermatocyte PDIA3 (*Stra8-Cre/Pdia3^fl/fl^)* and PDIA6 *(Stra8-Cre/Pdia6^fl/fl^)* were mated (Supplemental Figure 2). The *Stra8-Cre* gene induced the expression of Cre enzyme in the type A1 spermatogonium cells 3 days after birth and reached the peak expression at 7 days (20). With the initiation of the Cre enzyme, PDI was not expressed in germ cells, and this was confirmed by immunofluorescence and Western blotting in testicular tissue (Figure 2, C and D). The male *Stra8-Cre/Pdi^fl/+^* mice had normal fertility (Supplemental Figure 3). *Stra8-Cre/Pdi^fl/fl^* mouse survived normally after birth, with normal body size, appearance, and sex ratio. With increasing weekly age, the testicular size of *Stra8-Cre/Pdi^fl/fl^* male mice was gradually decreased, and the testicular body weight ratio was significantly reduced (Figure 2E). Male *Stra8-Cre/Pdi^fl/fl^* mice were mated with *Pdi^fl/fl^* female mice at a ratio of 1:2, and the litter birth of female mice was observed and recorded. *Pdi^fl/fl^* female mice appeared to have vaginal plugs but mated continuously for 3 months without offspring (Figure 2F), suggesting that testicle-specific PDI–KO leads to infertility in male mice. The H&E staining of testes showed that, in both *Stra8-Cre/Pdia3^fl/fl^* and *Stra8-Cre/Pdia6^fl/fl^* mice, spermatogenic tubule lumens were filled with spermatocytes, round spermatozoa, and elongated spermatozoa, without vacuole phenomena (Supplemental Figure 2A). The *Stra8-Cre/Pdia3^fl/fl^* and *Stra8-Cre/Pdia6^fl/fl^* mice likely had normal sperm counts in epididymis, shown by the H&E staining (Supplemental Figure 2B). These data suggest that PDI is selectively required for spermatogenesis.

PDI is the prototype member of this family and plays a vital role in various physiological conditions (21). We detected high expression of PDI in the liver, testes, and epididymis (Supplemental Figure 4A). At 2, 3, and 7 weeks, PDI expression was relatively high in all cell types, as shown by immunofluorescence staining (Supplemental Figure 4B). Moreover, in the testes of mice aged from 1 to 6 weeks, there was a rapid increase in the expression levels of PDI from 2 weeks until they reached sexual maturity (Supplemental Figure 4C), consistent with its involvement in male fertility.

**Figure 3:**
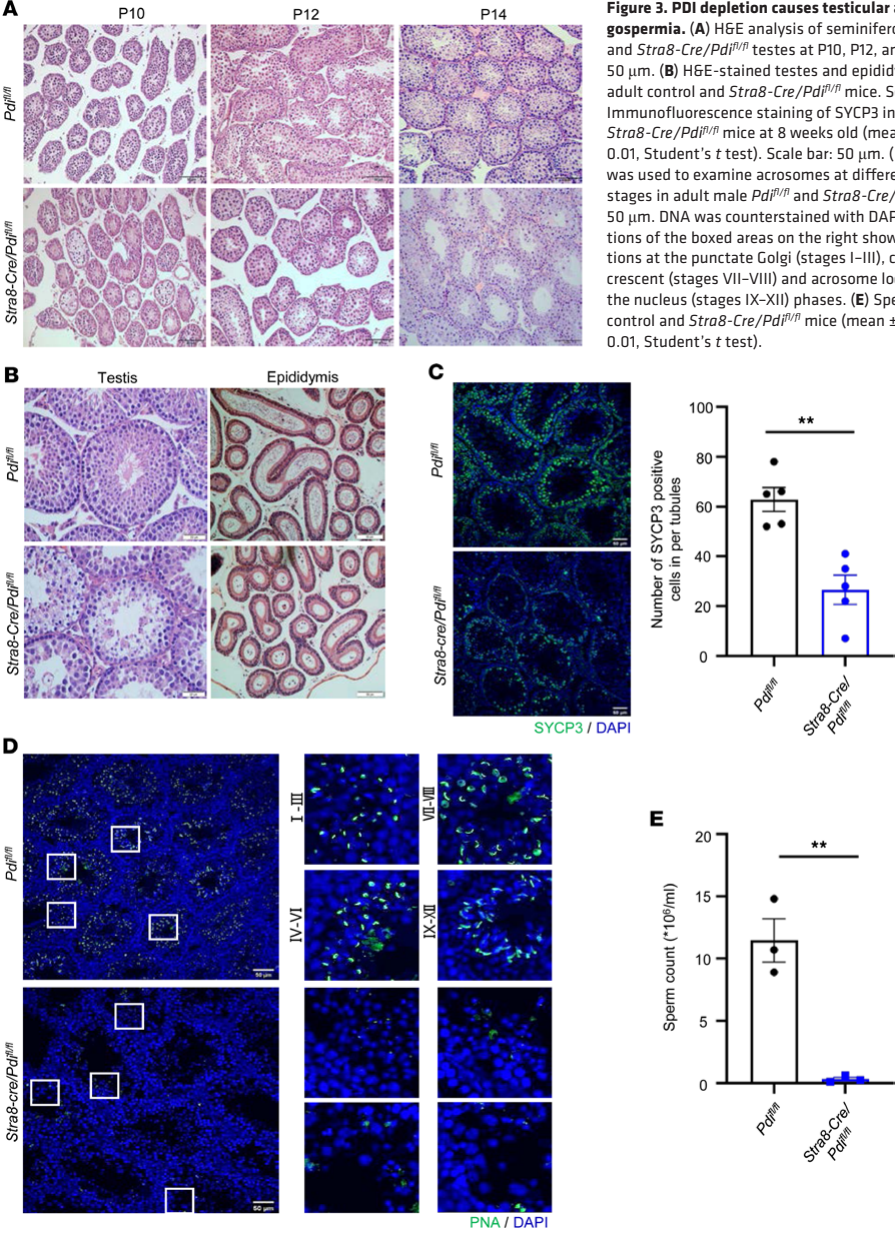
PDI depletion causes testicular atrophy and oligospermia. (**A**) H&E analysis of seminiferous tubules of control and *Stra8-Cre/Pdi^fl/fl^* testes at P10, P12, and P14. Scale bars: 50 μm. (**B**) H&E-stained testes and epididymal sections in adult control and *Stra8-Cre/Pdi^fl/fl^* mice. Scale bars: 50 μm. (**C**) Immunofluorescence staining of SYCP3 in testes of control and *Stra8-Cre/Pdi^fl/fl^* mice at 8 weeks old (mean ± SEM, *n* = 3, **P < 0.01, Student’s *t* test). Scale bar: 50 μm. (**D**) FITC-labeled PNA was used to examine acrosomes at different developmental stages in adult male *Pdi^fl/fl^* and *Stra8-Cre/Pdi^fl/fl^* mice. Scale bar: 50 μm. DNA was counterstained with DAPI. Higher magnifications of the boxed areas on the right show tubule cross-sections at the punctate Golgi (stages I–III), cap (stages IV–VI), and crescent (stages VII–VIII) and acrosome located at the top of the nucleus (stages IX–XII) phases. (**E**) Sperm counts from adult control and *Stra8-Cre/Pdi^fl/fl^* mice (mean ± SEM, *n* = 3, **P < 0.01, Student’s *t* test).

To understand the cause of infertility in *Stra8-Cre/Pdi^fl/fl^* male, we next examined the first wave of spermatogenesis at P10, P12, and P14 corresponding to leptotene, zygotene, and pachytene spermatocytes, respectively (22). The testes of the control *Pdi^fl/fl^* mice were filled with various stages of spermatocytes, but the seminiferous tubules of *Stra8-Cre/Pdi^fl/fl^* mice contained only a few spermatocytes without an obvious change in number (Figure 3A), suggesting that PDI deficiency affected the prophase of meiosis at the first wave of spermatogenesis in mice. In the adult mouse testes, the cells of *Stra8-Cre/Pdi^fl/fl^* at all levels were poorly developed, and many vacuoles appeared in the seminiferous tubules (Figure 3B). We analyzed early meiotic cells in testes sections after staining with synaptonemal complex protein 3 (SYCP3), typically present in meiotic prophase I spermatocytes, and we found that the number of prophase I spermatocytes was significantly reduced in *Stra8-Cre/Pdi^fl/fl^* testes (Figure 3C). FITC-labeled peanut agglutinin (PNA) was used to evaluate the acrosome development (23, 24). In the spermatogenic tubules of *Pdi^fl/fl^* mice, typically punctated Golgi bodies (stages I–III), capped acrosomes (stages IV–VI), or crescent-shaped acrosomes (stages VII–VIII) were observed in round spermatid acrosomes, and elongated spermatid acrosomes were located on top of the nucleus (stages IX–XII). However, there was no positive signal of FITC-PNA in *Stra8-Cre/Pdi^fl/fl^* mice and almost no spermatid in the testes (Figure 3D). The H&E staining of epididymis showed there was a markedly decreased sperm count in *Stra8-Cre/Pdi^fl/fl^* mice. The sperm count of *Pdi^fl/fl^* male mice was 11.47 × 10^6^ ± 1.74 × 10^6^/mL, while that of *Stra8-Cre/Pdi^fl/fl^* male mice was only 0.32 × 10^6^ ± 0.15 × 10^6^/mL (Figure 3E). Therefore, testicle-specific PDI deficiency leads to infertility in male mice due to the defect in the progress of the first wave of spermatogenesis, following oligospermia.

### PDI deficiency disrupts the meiosis of spermatocytes.

**Figure 4:**
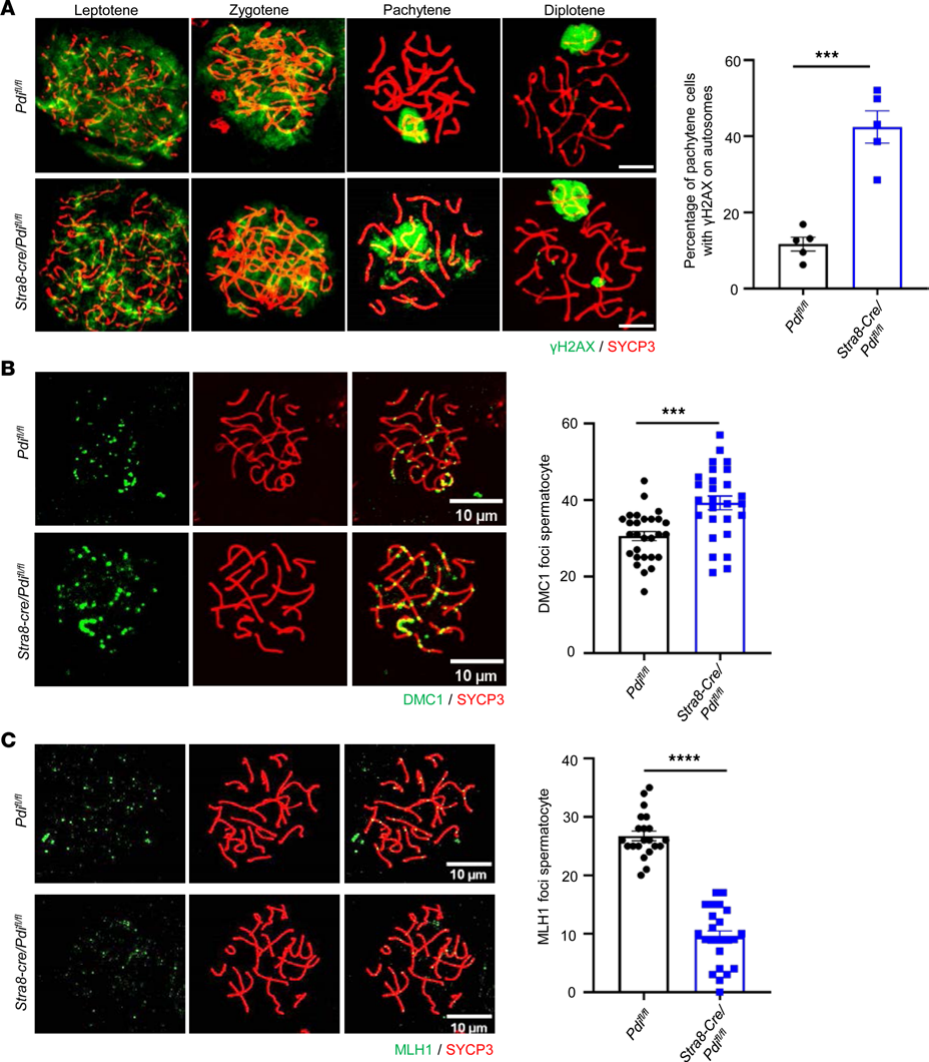
PDI deficiency disrupts the meiosis of spermatocytes. (**A**) Immunostaining of meiotic spermatocyte from adult control and *Stra8-Cre/Pdi^fl/fl^* mice for SYCP3 (red) and γH2AX (green). Percentage of pachytene spermatocytes that contained γH2AX foci on their autosomes (mean ± SEM, *n* = 5; 300 cells, ***P < 0.001, Student’s *t* test). Scale bar: 10 μm. (**B**) Immunostaining for SYCP3 (red) and DMC1 (green) in control *Pdi^fl/fl^* and *Stra8-Cre/Pdi^fl/fl^* spermatocytes. Quantification of average DMC1 foci per spermatocyte (mean ± SEM, n = 28 for *Pdi^fl/fl^*, *n* = 27 for *Stra8-Cre/Pdi^fl/fl^*, ***P < 0.001, Student’s *t* test). Scale bar: 10 μm. (**C**) Immunostaining for SYCP3 (red) and MLH1 (green) in control and *Stra8-Cre/Pdi^fl/fl^* spermatocytes. Quantification of average MLH1 foci per spermatocyte (mean ± SEM, n = 21 for *Pdi^fl/fl^*, *n* = 23 for *Stra8-Cre/Pdi^fl/fl^*, ****P < 0.0001, Student’s *t* test). Scale bar: 10 μm.

To further examine the role of PDI in meiosis, the prophase of meiotic passage by chromosomal spreads from the testes of mice was assessed and coimmunostained with SYCP3 and γH2AX (Figure 4A). All stages from leptotene to diplotene were observed in spermatocytes in both control and *Stra8-Cre/Pdi^fl/fl^* mice. When DNA double-strand breaks (DSBs) occurred, γH2AX signals were observed throughout the nucleus in leptotene and zygotene. When the autosomal breaks were repaired, γH2AX signals were restricted to the sex chromosome in pachytene and diplotene in control *PDI^fl/fl^* mice. Nevertheless, unrepaired DSBs with γH2AX signals partially retained on autosomes occupied 42.44% ± 4.23% of *Stra8-Cre/Pdi^fl/fl^* pachytene, which was markedly higher than 11.68% ± 1.80% of the control. In general, meiosis recombination defect is an important cause of pachytene retardation (25). Meiosis recombination depends on the successful repair of the program DSBs. Proteins were involved in the formation and recombination of meiotic-specific programmed DSBs, such as SYCP1, MRE11, RAD51, DMC1, and MLH1. With the help of RAD51 and DMC1 recombinases, the annealed DSB terminal develops into an extended D-loop (26), and the DSB is then repaired (27, 28). MLH1 represents the crossover during meiosis recombination (29). The different stages of DSBs processing in chromosome spreads of pachytene spermatocytes were further observed by coimmunostaining SYCP3 with MLH1 and DMC1. The positive signals of spermatocytes were counted in the pachytene stage. We found that the number of DMC1 loci in spermatocytes of *Stra8-Cre/Pdi^fl/fl^* mice was significantly increased compared with control *Pdi^fl/fl^* mice (Figure 4B), suggesting that the PDI deletion results in an increased incidence of unrepaired DSBs in spermatocytes during the pachytene stage. In addition, *Stra8-Cre/Pdi^fl/fl^* mice had significantly fewer MLH1 loci in spermatocytes than control *Pdi^fl/fl^* mice (Figure 4C), suggesting that cross-formation is also impaired. Thus, PDI is indispensable in meiosis and plays a pivotal role in DSB repair and synapsis in spermatocytes.

### PDI deficiency induces ER stress and apoptosis of spermatocytes.

**Figure 5:**
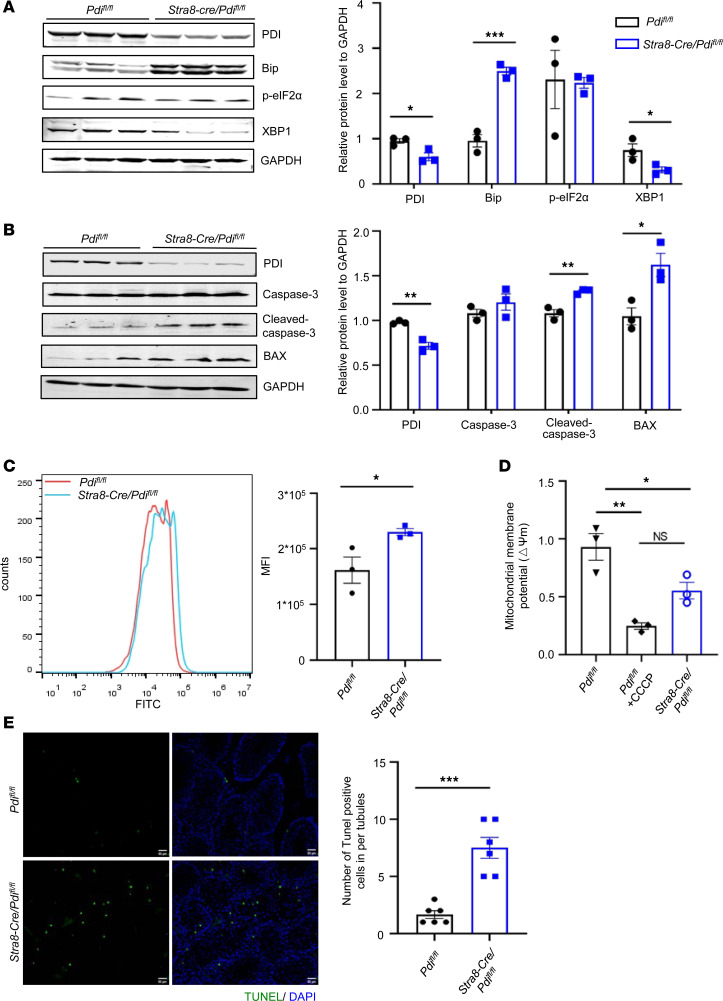
PDI deficiency induces ER stress and apoptosis of spermatocytes. (**A** and **B**) Western blotting of ER stress–related and apoptosis-related molecules in testes from adult control and *Stra8-Cre/Pdi^fl/fl^* mice. GAPDH as an internal control (mean ± SEM, *n* = 3, *P < 0.05, **P < 0.01, ***P < 0.001, Student’s *t* test). (**C**) Flow cytometric analysis of the ROS levels in spermatocytes enriched from adult control and *Stra8-Cre/Pdi^fl/fl^* mice (mean ± SEM, *n* = 3, *P < 0.05, Student’s *t* test). (**D**) JC-1 staining assay of spermatocytes enriched from adult control and *Stra8-Cre/Pdi^fl/fl^* mice (mean ± SEM, *n* = 3, *P < 0.05, **P < 0.01, 1-way ANOVA). (**E**) TUNEL assay of testes seminiferous tubules from control and *Stra8-Cre/Pdi^fl/fl^* at 8 weeks old (mean ± SEM, *n* = 3, ***P < 0.001, Student’s *t* test). Scale bar: 50 μm.

PDI is critical for correcting protein folding of nascent peptides by catalyzing the formation of disulfide bonds. The decrease in its expression often causes the accumulation of misfolded proteins beyond a specific limit leading to ER stress and unfolded protein response (UPR). In contrast, excessive ER stress may lead to apoptosis if the dysfunction of PDI cannot be compensated (30). Compared with the control group, the level of Bip (also known as GRP78) in *Stra8-Cre/Pdi^fl/fl^* testes was significantly increased (Figure 5A), demonstrating the presence of severe ER stress. In addition, *Stra8-Cre/PDI^fl/fl^* testes had a significant increase in Cleaved caspase-3 and key proapoptotic protein BAX (Figure 5B), suggesting the activation of the apoptosis signaling pathway. Consistent with the fact that the apoptosis signaling pathway is activated by high levels of intracellular reactive oxygen species (ROS) causing damage to proteins, nucleic acids, lipids, membranes, and organelles (31), *Stra8-Cre/Pdi^fl/fl^* spermatogenic cells had a significant increase in ROS (Figure 5C). JC-1 is an ideal fluorescent probe for detecting mitochondrial membrane potential (MMP) and is often used to evaluate apoptosis. The MMP of *Stra8-Cre/Pdi^fl/fl^* spermatogenic cells was decreased compared with that of *Pdi^fl/fl^* mice, suggesting the apoptosis of cells (Figure 5D). TUNEL staining of the testicular sections showed a significant increase in apoptotic cells in seminiferous tubules of *Stra8-Cre/Pdi^fl/fl^* mice (Figure 5E). The above data indicate that the absence of PDI induces irreversible ER stress and cells apoptotic in spermatocytes.

### PDI is required for the expression of HSPA4L, SHCBP1L, and DDX4 in spermatogenic cells.

**Figure 6:**
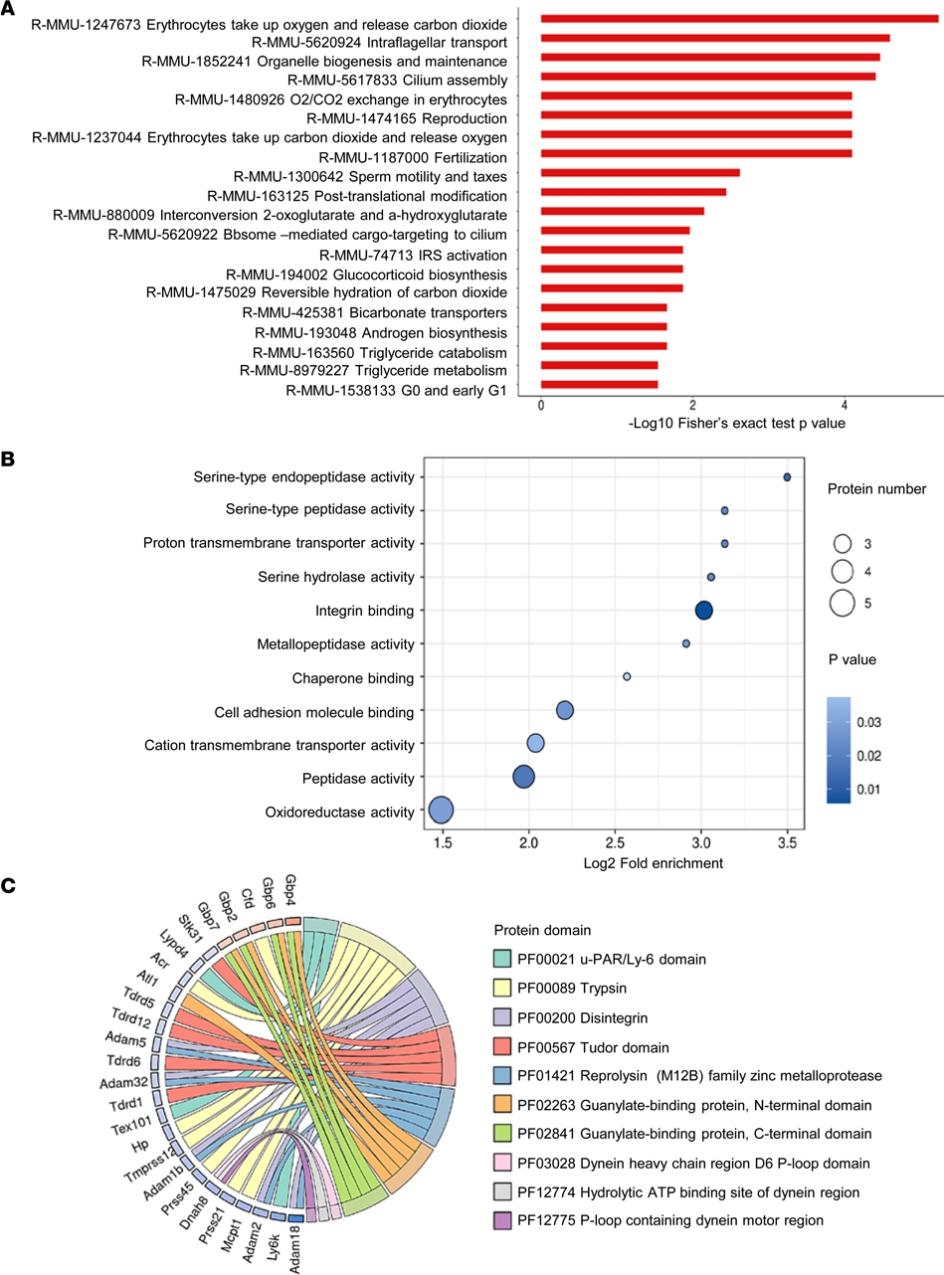
Identification of protein profiles in mouse testes altered by PDI deficiency. (**A**–**C**) Reactome, gene ontology, and protein domain analysis of downregulated and upregulated genes in control *Pdi^fl/fl^* and *Stra8-Cre/Pdi^fl/fl^* testes.

**Figure 7:**
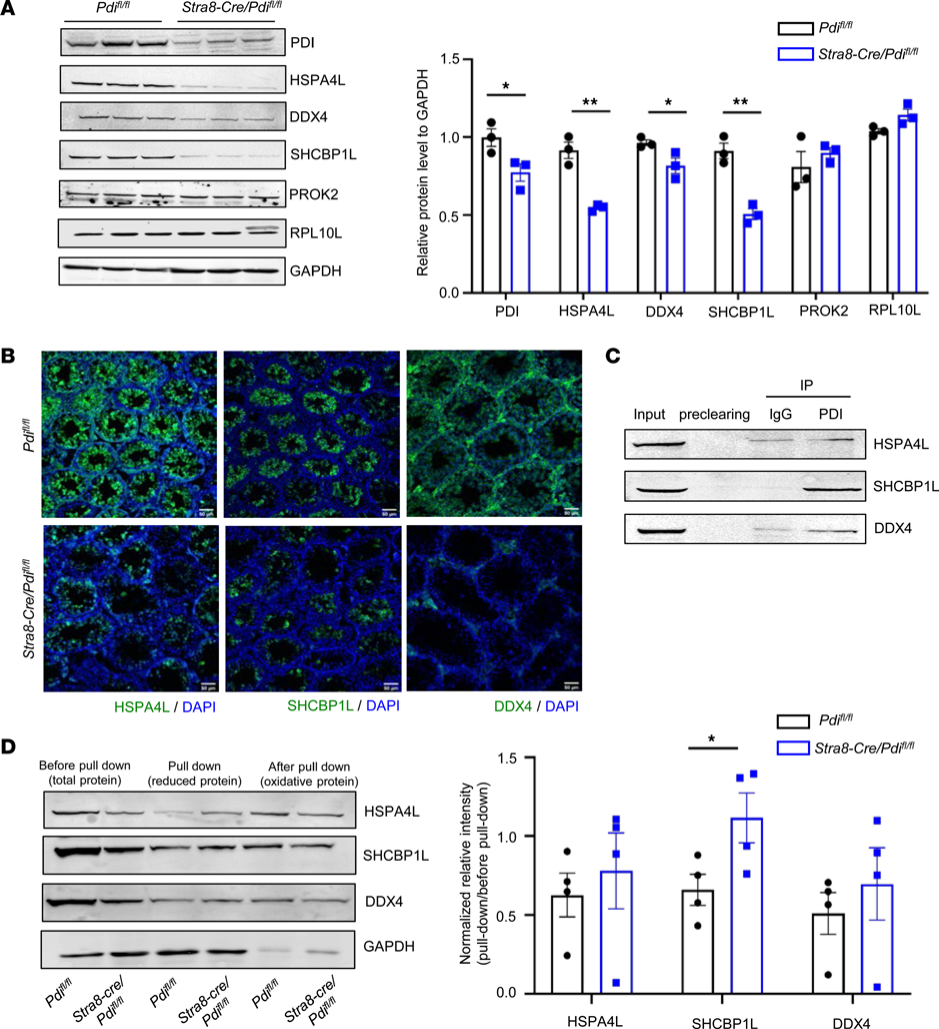
PDI is required for the expression of HSPA4L, DDX4, and SHCBP1L in spermatogenic cells. (**A**) Western blotting analysis of proteins in testes from adult control and *Stra8-Cre/Pdi^fl/fl^* mice, normalized to GAPDH (mean ± SEM, *n* = 3, *P < 0.05, **P < 0.01, Student’s *t* test). (**B**) Immunofluorescence staining of HSPA4L, DDX4, and SHCBP1L in testes from control and *Stra8-Cre/Pdi^fl/fl^* mice at 3 weeks old. Scale bar: 50 μm. (**C**) Immunoprecipitation of testes lysates of WT mice was performed with anti-PDI antibody. Detection of HSPA4L, DDX4, and SHCBP1L in anti-PDI immunoprecipitants with Western blotting. (**D**) *Pdi^fl/fl^* and *Stra8-Cre/Pdi^fl/fl^* testes lysates were labeled with MPB, and the labeled proteins were pulled down by streptavidin beads and analyzed with Western blotting using the indicated antibodies. The relative abundance of reduced protein was calculated by comparing the density of protein before and after the pull-down, and then the value of the *PDI^fl/fl^* group was normalized to be 1 (mean ± SEM, *n* = 4, *P < 0.05, Student’s *t* test).

To identify the potential substrates of PDI in male reproduction, we used 4D-Fast DIA-based quantitative proteome measurement of protein expression profiles of testes in control *Pdi^fl/fl^* mice and *Stra8-Cre/Pdi^fl/fl^* mice at P14. The Four-dimensional (4D) fast data-independent acquisition–based (DIA-based) proteomics method indicates a total of 193 upregulated proteins and 341 downregulated proteins with 1.5-fold or more between the 2 groups. Found by analysis through GO (http://geneontology.org), KEGG (https://www.kegg.jp), Domain (http://pfam.xfam.org), Reactome (https://reactome.org), and WiKipathways (https://www.wikipathways.org) as well as published literature, most of the proteins were related to spermatogenesis, sperm morphological function, apoptosis, and cell adhesion; these proteins included AXDND1 (32), FANCA (33), HSF5 (34), HSPA4L (3), MRNIP (35), RPL10L (36), SHCBP1L (4), TMPRSS12 (37), SOX30 (38), and DDX4 (5) (Figure 6, A–C). Western blotting was used to verify the expression levels of these proteins, showing that the expression of HSPA4L, SHCBP1L, and DDX4, at least, were decreased in *Stra8-Cre/Pdi^fl/fl^* testes. However, the expression of PROK2 and RPL10L showed no difference (Figure 7A). Because PDIA3, PDIA4, PDIA5, PDIA6, PDIA9, PDIA14, PDIA15, and PDIA16 were expressed in testes, as shown in Figure 1A, we measured the expression of HSPA4L, SHCBP1L, and DDX4 in these gene deficient mice. Western blotting showed that the expression of HSPA4L, SHCBP1L, and DDX4 in testes of *Stra8-Cre/Pdia3^fl/fl^*, *Pdia4^–/–^*, *Pdia5^–/–^*, *Stra8-Cre/Pdia6^fl/fl^*, *Pdia9^–/–^*, *Pdia14^–/–^*, *Pdia15^–/–^*, and *CAG-Cre/Pdia16^fl/fl^* mice were comparable with their control mice (Supplemental Figure 5, A–H). All of these KO mice had comparable PDI expression levels in the testicular lysate compared with the control mice, especially the mice lacking PDIA3 and PDIA6 whose structure and amino acid homology are close to PDI. The expression of HSPA4L, SHCBP1L, and DDX4 was selectively decreased by PDI deficiency, consistent with the observations of immunofluorescence staining showing the decreased expression of HSPA4L, SHCBP1L, and DDX4 in *Stra8-Cre/Pdi^fl/fl^* testes (Figure 7B). Moreover, in testicular lysate, the physical association of PDI with HSPA4L, SHCBP1L, and DDX4 was detected by coimmunoprecipitation (Figure 7C). These data suggest the specific crucial role of PDI in expression of these proteins, and it likely underlies the phenotypes of PDI deficiency. The most important function of the PDI family is to help the correct folding of the new protein disulfide bonds in the ER, to maintain the proteostasis. To assess the potential role of PDI, free thiols of proteins in the lysate of testes from *Pdi^fl/fl^* and *Stra8-Cre/Pdi^fl/fl^* mice were labeled with 3-(N-maleimide-propionyl)-biocytin (MPB), and the MPB-labeled proteins were pulled down by streptavidin beads. The immunoblotting results revealed more abundant free thiols of SHCBP1L in PDI-deficient testes lysate than that of the control, although the total SHCBP1L protein was much less in PDI-deficient testes lysate (Figure 7D). The ratio of normalized levels of MPB-labeled SHCBP1L/total SHCBP1L shows that PDI deficiency increased free thiols in SHCBP1L, suggesting that PDI serves as a thiol oxidase that is responsible for the disulfide bond formation of SHCBP1L. However, the free thiols of SHCBP1L in the lysate of testes were comparable between *Pdia3^fl/fl^* mice and *Stra8-Cre/Pdia3^fl/fl^* mice (data not shown). Although PDI deficiency did not change free thiols in HSPA4L and DDX4 proteins, we have to admit the shortage/limitation of this MPB labeling technique, as it could not identify the disulfide isomerization reaction. Taken together, our data imply that PDI plays an essential role in spermatogenesis, at least in part, through the regulation of HSPA4L, SHCBP1L, and DDX4 expressions in spermatocytes.

## Discussion

In the present study, using a group of gene-KO mice deficient in 14 members of the PDI family, we found that only PDI deficiency caused the defect in sperm production, uncovering an essential role of PDI in spermatogenesis. The phenotype of sperm production caused by PDI deficiency was confirmed by inducible *UBC-Cre*–driven whole-body and spermatocyte-specific *Stra8-Cre* gene–KO strains; in both, PDI was completely deleted without affecting the expression of other members of the PDI family. Furthermore, we found that PDI deficiency induced ER stress and impaired meiosis and apoptosis of spermatocytes, consistence with a marked reduction in sperm counts and complete male infertility. The proteomic analysis using 4D-FastDIA indicates the altered profiles of proteins in testes between the littermate control *Pdi^fl/fl^* mice and *Stra8-Cre/Pdi^fl/fl^* mice, indicating that the critical proteins in meiosis, such as AXDND1, FANCA, HSF5, HSPA4L, MRNIP, RPL10L, SHCBP1L, TMPRSS12, SOX30, and DDX4, were significantly downregulated. Taken together, our study demonstrates an essential role for PDI in the proteostasis of spermatocytes to support meiosis.

PDI is one of the most abundant and critical protein-folding catalysts in the ER of eukaryotic cells (30). Its a and a′ domains have catalytic activity, while the b and b′ domains have substrate binding activity (7). Although PDI has more than a single function in a variety of cellular processes and is responsible for the folding and synthesis of microsomal triglyceride transfer protein, insulin, and P4H, which are critical for lipid and glucose metabolism and bone formation (39–41), its role in spermatogenesis has never been studied, to our knowledge, using gene-modified models. A previous study has shown that mice lacking PDIA7 had a defect in male fertility due to loss of sperm migration from the uterus into the oviduct because PDIA7 deletion reduced the expression of ADAM3. However, PDIA7 deficiency did not affect spermatogenesis, and sperm produced were morphologically normal (12). Our data indicate that PDI and other PDI family members PDIA3, PDIA6, and PDIA7 were highly expressed in testes (Figure 1A), consistent with previous studies. PDIA3 and PDIA6 have similar structures to PDI and both contain 2 CGHC active domains. However, the deficiency of PDIA3 and PDIA6 as well as the other 11 members of this family did not cause the defect in spermatogenesis (Supplemental Figures 1 and 2), suggesting that PDI is selectively required for this process and its function is indispensable.

The major function of PDI is to catalyze the disulfide bond formation of native proteins in ER, and this is necessary for normal protein conformation to maintain their function. If PDI is deleted or has low activity, a large number of misfolded proteins accumulate in the ER, leading to ER stress, increased intracellular ROS, and decreased protein synthesis. In this study, we found that ER stress marker Bip was highly upregulated together with the activation of UPR activation pathways involving XBP1 (Figure 5A). These results suggest that PDI deficiency causes severe ER stress and UPR. However, the deleterious effect of PDI deficiency cannot be compensated for by any other PDI enzymes, leading to persistent intracellular ROS elevation and triggering proapoptotic pathways (Figure 5, B–D). Therefore, PDI is a critical component of proteostasis in spermatocytes. It is estimated that approximately 10%–20% of couples cannot conceive a child in the world (42). Male factor infertility is estimated at 30%–50% of infertility cases (43). Azoospermia, or severe oligospermia, is a more serious condition of male infertility caused by multiple factors such as genetic, epigenetic, and environmental factors. Genetic factors may account for about 15%–30% of cases of male infertility, with over 2,000 genes devoted to spermatogenesis (44). Our finding on the essential role of spermatogenesis will boost further investigation to understand whether the decreased expression and enzymatic activity of PDI is involved in male infertility.

Using a high-throughput technique, we found that PDI deficiency caused a significant decrease of 341 proteins with a ≥1.5-fold change, among which AXDND1, FANCA, HSF5, HSPA4L, MRNIP, RPL10L, SHCBP1L, TMPRSS12, SOX30, and DDX4 have been shown having a role in spermatogenesis. Using Western blotting, we have so far confirmed the decreased expression of HSPA4L, SHCBP1L, and DDX4 (Figure 7A). As found in a previous study, in *Hspa4l^–/–^* mice, spermatogenic tubules underwent apoptosis and their mature sperm number was significantly reduced (3). *Shcbp1l^–/–^* mice exhibited an increase in metaphase or anaphase-arrested spermatocytes, as well as impaired fertility in male mice (4). DDX4 KO also resulted in male infertility and their proliferative activity of primordial germ cells was reduced; DDX4-null spermatogonia failed to reach the fertilized egg stage and undergo apoptosis (5). These proteins are necessary for spermatogenesis and fertility. The decreased expression of all of these proteins may underlie the phenotypes of PDI deficiency and suggest a role of PDI in the proteostasis of spermatocyte ER that is crucial for correct folding and synthesis of these critical proteins. Presumably, PDI is required for the formation of structural disulfide bonds of HSPA4L, SHCBP1L, and DDX4; the deficiency of PDI might cause the unfolded or misfolded structure of these proteins, leading to their degradation. To understand the specific role of PDI in regulation of these proteins, it will be helpful to identify the cysteines/disulfides targeted by PDI in these proteins. Mouse and human HSPA4L have 15 and 18 Cys residues, respectively (45). Both mouse and human SHCBP1L have 13 Cys residues. There are 15 Cys residues in mouse and human DDX4. However, the crystal structure of these proteins has not been characterized so far, their disulfide pairings are not known. In this case, we studied the oxidation of protein by PDI using the thiol labeling technique. We found the association of PDI deficiency with the increase in free thiols of these proteins, suggesting that PDI catalyzes the disulfide oxidation of these proteins. Our observations point out the need to characterize the disulfide pairing of these proteins so that we will be able to map the targeting cysteines of PDI using new techniques, such as differential thiol labeling and mass spectrometry (MS).

In summary, our data provide genetic evidence demonstrating that PDI plays an essential role in spermatogenesis and male fertility and that its function in meiosis is indispensable. Our findings highlight the importance of spermatocyte proteostasis and an ER quality control system for spermatogenesis as well as the distinct requirement of different PDI family members in this process. In the future, it will be necessary to characterize the disulfide pairing of the key molecules in spermatogenesis for a better understanding of the thiol-based redox network driven by PDI in the physiological and pathophysiological settings.

## Methods

### Sex as a biological variable.

Our study used male mice because the phenotype of spermatogenesis is exclusively relevant in males.

### Mice.

*Stra8-Cre/Pdi^fl/fl^* mice were generated by mating *Stra8-Cre* mice with *Pdi^fl/fl^* mice. *Stra8-Cre* mice were provided by Minghan Tong from the State Key Laboratory of Molecular Biology in Shanghai. *UBC-Cre/Pdi^fl/fl^* mice were generated by mating *UBC-Cre* mice with *Pdi^fl/fl^* mice. *UBC-Cre* mice were purchased from Shanghai Model Organisms Center Inc. The embryonic stem cells for PDI-floxed mice (clone EPD0317_6_D10), PDIA2 (PDIp) KO first (*Pdia2^–/–^*) mice (clone EPD0753_5_D11), PDIA5 (PDIr) KO first (*Pdia5^–/–^*) mice (clone DEPD00576_3_G10), PDIA6-floxed mice (clone HEPD0530_2_F02), PDIA8 (ERp27) KO first (*Pdia8^–/–^*) mice (clone EPD0688_4_C05), PDIA9 (ERp29) KO first (*Pdia9^–/–^*) mice (clone EPD0667_5_E05), PDIA13 (TMX3) KO first (*Pdia13^–/–^*) mice (clone HEPD0721_1_D05), PDIA14 (TMX4) KO first (*Pdia14^–/–^*) mice (clone EPD0684_1_C05), PDIA15 (ERp46) KO first (*Pdia15^–/–^*) mice (clone HEPD0564_9_A11), and PDIA19 (ERdj5) KO first (*Pdia19^–/–^*) mice (clone HEPD0507_5_B06) were generated by the International Knockout Mouse Consortium (IKMC) at the Cambridge-Suda Genomic Resource Center. After passing production quality control, the ES cells were injected into murine blastocysts and transferred to pseudopregnant female mice to generate the target KO first mice. PDIA16 (ERp18) whole-body KO mice (*CAG-Cre/Pdia16^fl/fl^*) were generated by mating CAG-Cre mice with *Pdia16^fl/fl^* mice, which were produced by a CRISPR/Cas9-based protocol (Cyagen Biosciences Inc.) Briefly, the gRNA to mouse *Pdia16* gene, the donor vector containing loxP sites, and Cas9 mRNA were coinjected into fertilized mouse eggs to generate targeted conditional KO offspring. PDIA4 KO first (*Pdia4^–/–^*) mice (15), PDIA11 KO first (*Pdia11^–/–^*) mice (16), and *Pdia3^fl/fl^* mice (14) were described in our previous studies. Spermatocyte-specific–KO mice deficient in PDI, PDIA3, and PDIA6 were generated by mating floxed mice with *Stra8-Cre* mice. Genotyping of mice was performed by PCR analysis of tail DNA, and primers used for genotyping are listed in Supplemental Table 1.

### Inducible PDI–KO with UBC-Cre/Pdi^fl/fl^ mice.

To generate tamoxifen-inducible PDI–KO mice, *UBC-Cre/Pdi^fl/fl^* mice were treated with tamoxifen as described previously (19). Briefly, 7-week-old male mice received i.p. injection of 100 μg/g/day tamoxifen (Sigma-Aldrich, T5648) each day for 7 days.

### qPCR.

Mouse testes (20–50 mg) were homogenized in 1 mL Trizol reagent (Vazyme, R411-01), and total RNA was extracted. First-strand cDNA synthesis was performed using the 1st-strand cDNA Synthesis Kit (Vazyme, R111-01) according to the instructions of the manufacturer. Reverse transcription PCR (RT-PCR) was performed on a 7500 Real-time PCR system (Applied Biosystems) using a SYBR qPCR Master Mix (Vazyme, Q712-02). The relative quantitative expression of PDIs was calculated as log(2^–ΔΔCt^). The primers are listed in Supplemental Table 2.

### Western blotting and immunoprecipitation.

Mice were euthanized, and the testes were perfused with phosphate-buffered saline (PBS). The testes were lysed with lysis buffer (20 mM Tris-HCl, 150 mM NaCl [pH 7.4], 1% Triton X-100, 1 mM EDTA, 0.5% sodium deoxycholate) containing protease inhibitor cocktail (Thermo Fisher Scientific, 89900). Protein concentration was measured using a BCA Protein Assay Kit (Beyotime, P0011). Equal amounts of testes lysates were resolved by SDS-PAGE and transferred onto a polyvinylidene difluoride (PVDF) membrane. The membrane was blocked by 5% skim milk for 1 hour at room temperature and probed with various antibodies as indicated. For immunoprecipitation, the antibody was added into testes lysates and incubated overnight at 4°C, followed by incubation with protein G beads (Genscript, L00209) for 2 hours. The beads were washed with lysis buffer 3 times and boiled with 2 × Laemmli sample buffer (Bio-Rad, 1610737) containing 5% β-mercaptoethanol for 5 minutes at 100°C. The samples were separated by SDS-PAGE and detected by immunoblotting. Blots were incubated with specific antibodies against PDI, PDIA3, HSPA4L, and PROK2 (ABclonal; A19239, A1085, A17637, and A6705, respectively); PDIA2 (Invitrogen, PA5-112644), PDIA4, XBP1, and Bip (Cell Signaling Technology; 5033, 40435, and C50B12, respectively); PDIA5, PDIA14, PDIA15, DDX4, and SHCBP1L (Proteintech; 15545-1-AP, 21348-1-AP, 19834-1-AP, 51042-1-AP, and 27108-1-AP, respectively); PDIA6, PDIA8, and PDIA9 (Abcam; ab154820, ab181172, and ab11420, respectively); PDIA16 (Novus Biologicals, SR1258); RPL10L (Erpantech, AB-07-1234); p-eIF2α (Thermo Fisher Scientific, RD226932); and Caspase-3, Cleaved caspase-3, and BAX (Abmart; TA6311, T61532, and T40051, respectively). GAPDH (Proteintech, 60004-1-Ig) was used as a loading control. The intensity of each band was quantitated using ImageJ software (NIH) and normalized to the loading control.

### Assessment of fertility.

Each adult *Stra8-Cre/Pdi^fl/fl^* male or littermate control *Pdi^fl/fl^* male mouse was mated with 2 adult female *Pdi^fl/fl^* mice for fertility tests. The number of pups per litter and the date of delivery were recorded. The fertility test lasted for at least 3 months.

### TUNEL assay.

Mouse testicular frozen sections were fixed with 4% paraformaldehyde for 30 minutes and incubated with PBS containing proteinase K for 20 minutes. After washing 3 times with PBS, the testes sections were incubated with an equilibration buffer for 20 minutes. The TUNEL assay was performed according to the manufacturer’s instructions (Service Bio, G1501). In brief, the sections were incubated with the TUNEL kit including TdT enzyme and dUTP at 37°C for 1 hour. Sections were washed with PBS and then counterstained with DAPI. Slides were imaged under an FV3000 Confocal laser scanning microscopy (Olympus).

### Histological and immunofluorescence staining analysis.

Histological and immunohistochemical staining were performed as previously described with slight modifications (46). For paraffin sectioning, testes were excised and fixed in 4% paraformaldehyde at 4°C overnight. After the samples were dehydrated with graded ethanol and were paraffin embedded, the specimens were sectioned at 5 μm and mounted on glass slides. The sections were then deparaffinized, hydrated, and stained with H&E. The slides were imaged with a Leica DM2000 microscope. For immunofluorescence staining, testes were fixed in 4% paraformaldehyde at 4°C overnight and then dehydrated with 20% sucrose solution overnight. The testes were dipped in OCT compound and then quickly frozen in liquid nitrogen–frozen sections with a thickness of 10 μm and fixed in precooled acetone for 20 minutes. The slides were blocked with Immunol staining blocking buffer (QuickBlock Blocking Buffer for Immunol Staining, Beyotime, P0260) for 1 hour before being incubated with primary antibodies overnight at 4°C. After washing in PBS 3 times, the sections were incubated with secondary antibodies at room temperature for 2 hours. The images were obtained with an Olympus microscope and processed with ImageJ (NIH). Immunofluorescence labeling was performed using anti-SYCP3 (Santa Cruz Biotechnology Inc., sc-74569), anti-γH2AX (Cell Signaling Technology, 5438), and anti-DMC1 and -MLH1 (Proteintech; 13714-1-AP, and 11697-1-AP).

### Sperm count and staining.

Epididymis was separated from adult male mice and placed into an EP tube containing 1 mL PBS. After incubation at 37°C for 20 minutes, the sperm dispersed spontaneously. For sperm counting, the sperm suspension was diluted, and the drops were added to the blood cell counter for counting.

### PNA staining of testes sections.

PNA was used to detect the outer acrosomal membrane of spermatids. After deparaffinization and rehydration, tissue sections were stained with FITC-conjugated PNA (Sigma-Aldrich, L7381) for 30 minutes at 37°C. DAPI was used to stain the nuclei.

### Enrichment of spermatogenic cells.

Spermatogenic cells were enriched using a 2-step enzymatic digestion process as previously described (47). Briefly, the testes were decapsulated, cut, and added with 2 mL PBS containing 0.1% collagenase (Sigma-Aldrich, C5138) and 1 mg/mL DNase (Solarbio, D8071). After incubation at 37°C for 30 minutes, the cell suspension was centrifuged at 300*g* for 3 minutes, and the supernatant was discarded. PBS containing 0.25% Trypsin was used to resuspend the precipitates, followed by incubation at 37°C for 20 minutes with shaking every 3–5 minutes. A small amount of suspension was taken every 5 minutes under the microscope to observe whether it had been digested into a single-cell state. PBS containing 10% FBS serum was added to terminate digestion, and the samples were filtered through a 100 μm mesh cell strainer. After centrifugation at 300*g* for 5 minutes, the cell pellet was suspended in 10 mL of the DMEM and seeded in a 10 cm culture dish. After 4–6 hours of incubation at 37°C, the floating and weakly adhering cells were collected.

### 4D-FastDIA–based quantitative proteome analysis of mouse testes.

Quantitative proteomics was performed based on liquid chromatography–tandem MS (LC-MS/MS) technology. Protein extraction, in-solution trypsin digestion, and LC-MS/MS analysis were performed as previously described (48). Briefly, the testes isolated from 3 pairs of *Stra8-Cre/Pdi^fl/fl^* and *Pdi^fl/fl^* mice were mixed and ground in liquid nitrogen. The samples were sonicated 3 times on ice after adding 4 volumes of lysis buffer (8M urea, 1% protease inhibitor cocktail, 3 μM trichostatin A, 50 mM nicotinamide, and 2 mM EDTA). After centrifugation at 12,000*g* for 10 minutes at 4°C, the supernatant was collected, and the protein concentration was measured. For digestion, the protein solution was reduced with 5 mM dithiothreitol (Sigma-Aldrich, D0632) at 56°C for 30 minutes and alkylated with 11 mM iodoacetamide (Sigma-Aldrich, I6125) for 15 minutes at room temperature in darkness. The sample was diluted by adding 100 mM TEAB to a urea concentration of less than 2M. Finally, trypsin was added at 1:50 trypsin/protein mass ratio for the first digestion overnight and 1:100 trypsin/protein mass ratio for a second 4-hour digestion. Finally, the peptides were desalted by the C18 SPE column. The tryptic peptides were dissolved in solvent A (0.1% formic acid, 2% acetonitrile/in water) and directly loaded onto a homemade reversed-phase analytical column (25 cm length, 75 μm i.d.). The peptides were subjected to a capillary source followed by the timsTOF Pro (Bruker Daltonics) MS. The electrospray voltage applied was 1.60 kV. Precursors and fragments were analyzed at the TOF detector, with an MS/MS scan range from 100 to 1700 *m/z*. The timsTOF Pro was operated in parallel accumulation serial fragmentation (PASEF) mode. The resulting MS/MS data were processed using the MaxQuant search engine (v.1.6.15.0). Bioinformatics methods were employed for analysis, including protein annotation and protein function enrichment based on cluster analysis box protein interaction network analysis.

### MMP assay.

JC-1 MMP detection kit (Beyotime, C2003S) was used to determine the MMP. Briefly, the enriched spermatogenic cell suspension was incubated with JC-1 staining buffer in the dark at 37°C for 30 minutes. After washing 3 times with PBS, the testicular cell suspension was analyzed by the Spectramax M5 plate reader (Molecular Devices). JC-1 monomers emit green fluorescence, while JC-1 aggregates produce red fluorescence. MMP was analyzed by relative ratio of red/green fluorescence.

### ROS measurement.

The ROS assay kit (Beyotime, S0033S) was used to determine the ROS of enriched spermatogenic cells of *Pdi^fl/fl^* and *Stra8-Cre/Pdi^fl/fl^* mice. Briefly, cells (1 × 10^6^) were resuspended in DCFH-DA at a final concentration of 10 μM, followed by incubation at 37°C for 30 minutes. After washing 3 times with serum-free cell culture medium to entirely remove DCFH-DA, the samples were detected by flow cytometry.

### Spermatocyte spreading.

Spermatocyte surface spreading assay of testes was performed as previously described (23). In brief, mouse testicles were cleaned with PBS, the tunica albuginea was removed, and the seminiferous tubules were placed in a hypoosmotic solution (30 mM Tris [Sangon Biotech, A501492], 50 mM sucrose [Sinopharm Chemical Reagen, 10021418], 17 mM citric acid [Sangon Biotech, A100529], 5 mM EDTA [Beyotime, ST066], 2.5 mM DTT [Sigma-Aldrich, D0632], 1 mM PMSF [Sigma-Aldrich, 93482], pH 8.2) at room temperature for 20 minutes. The tubules were placed in a sucrose solution on the slide and gently squeezed to make sure that the cells in the tubules were fully released. Cell suspensions were fixed with 1% paraformaldehyde containing 0.15% Triton X-100 for 6 hours. The slide was washed in a 0.4‰ Photo-Flo200 and dried at room temperature for 10 minutes. After blocking with Blocking Buffer for Immunol Staining (Beyotime, P0260) for 30 minutes, the slides were incubated with primary antibody at 37°C overnight, followed by washing with TBS and then being incubated with secondary antibodies at 37°C for 2 hours. The slides were covered with Vectashield Mounting Medium (Servicebio, G1401) and coverslips. Slides were viewed under an FV3000 Confocal laser scanning microscopy (Olympus).

### MPB labeling and pull-down assay.

The testes tissues isolated from *Pdi^fl/fl^* and *Stra8-Cre/Pdi^fl/fl^* mice were solubilized using lysis buffer (20 mM Tris-HCl [Sangon Biotech, A501492], 150 mM NaCl [Sinopharm Chemical Reagen, 10019308] [pH 7.4], 1% Triton X-100 [Solarbio, T8200], 1 mM EDTA [Beyotime, ST066], 0.5% sodium deoxycholate [Sigma-Aldrich, D6750], and protease inhibitor cocktail [Thermo Fisher Scientific, A32961]). The lysates were centrifuged at 500*g* to discard cell debris. Mouse testes lysates (2–3 mg/mL, 200 μL) were incubated with 25 μM of 3-(N-maleimide-propionyl) biocytin (MPB) (Sigma-Aldrich, HY-W020784) for 30 minutes at room temperature, followed by the addition of 100 μM of glutathione (GSH) (Solarbio, G8180) to quench the labeling reaction. The lysates were incubated with streptavidin agarose beads (Thermo Fisher Scientific, 20349) in rotation overnight at 4°C. After washing with lysis buffer, the beads were boiled with 80 μL of 2 × Laemmli sample buffer (Bio-Rad, 1610737) containing 5% β-mercaptoethanol for 5 minutes. The samples were analyzed by SDS-PAGE and Western blotting.

### Statistics.

The experiments were performed at least in triplicates for statistical analysis. Data analysis was performed using the GraphPad Prism 8 software. For parametric comparison, 1-way ANOVA for multiple groups and 2-tailed Student’s *t* test for 2 groups were used, and the values were expressed as the mean ± SEM. *P* < 0.05 was considered statistically significant.

### Study approval.

Experiments with mice were performed following the institutional guidelines and with the approval of the IACUC of Soochow University. Mice were housed in a specific pathogen–free (SPF) animal facility at Soochow University under a 12-hour light/dark cycle, a temperature range of 24°C ± 2°C, and a humidity of 55% ± 5%. Littermates were used as controls in experiments unless specified otherwise.

### Data availability.

Data are available in the Supporting Data Values XLS file.

## Author contributions

YZ, AY, ZZ, and XY performed the experiments, and FC, YH, and DW assisted with experimental performance and critical reagents. YZ, ZZ, DW, and YW conceived and designed the study and wrote the manuscript. The order of co–first authors was based on the timeline of contributions.

## Supplementary Material

Supplemental data

Unedited blot and gel images

Supporting data values

## Version 1 06/24/2024

Electronic publication
