# Supplementary material for: Protein disulfide isomerase is essential for spermatogenesis in mice
Source: JCI Insight. 2024 Jun 24;9(12):e177743. doi: 10.1172/jci.insight.177743 (PMC11383184; doi:10.1172/jci.insight.177743)
Supplement: Supplemental data [file jciinsight-9-177743-s260.pdf]

Supplemental Figure 1

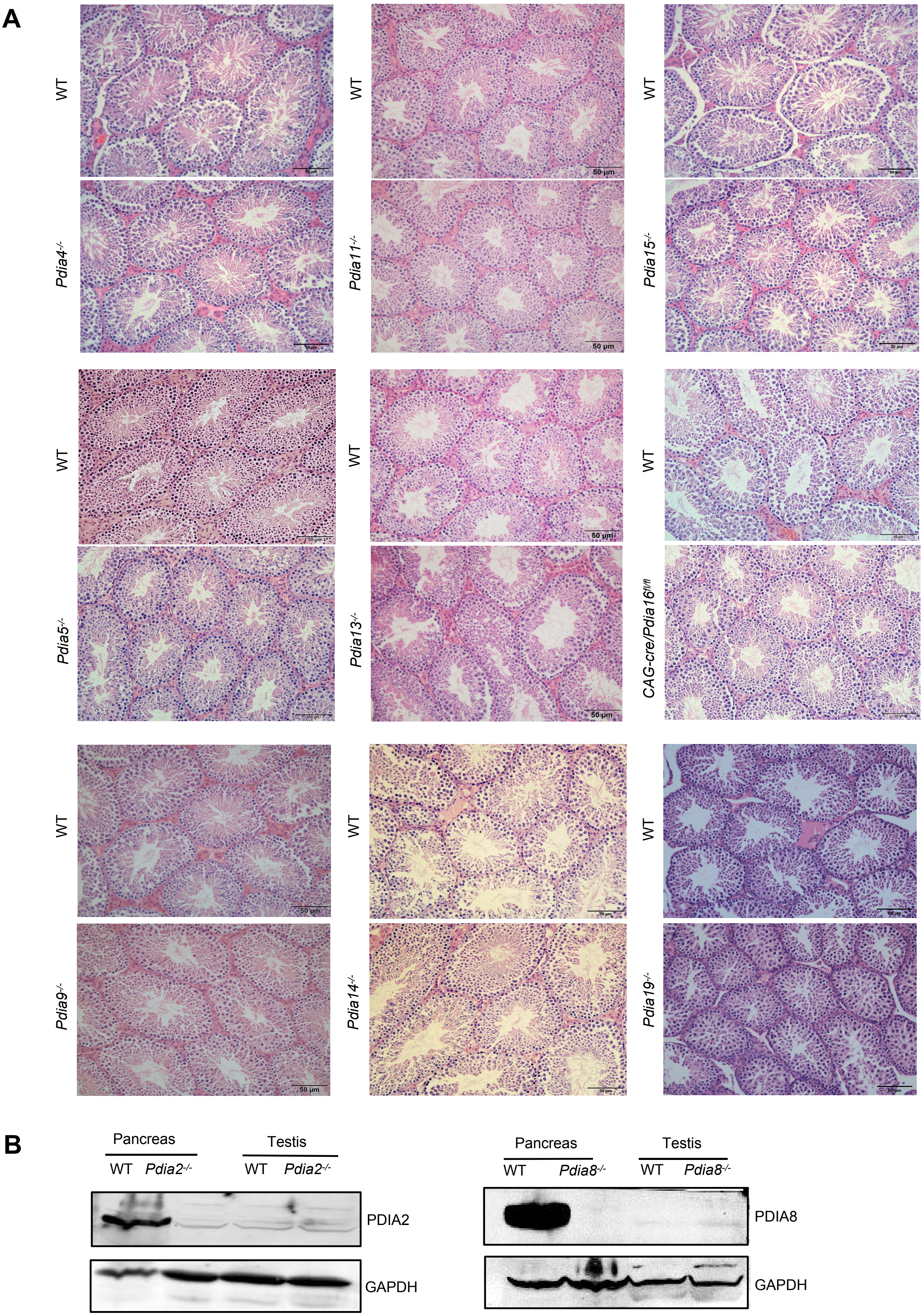

Supplemental Figure 2

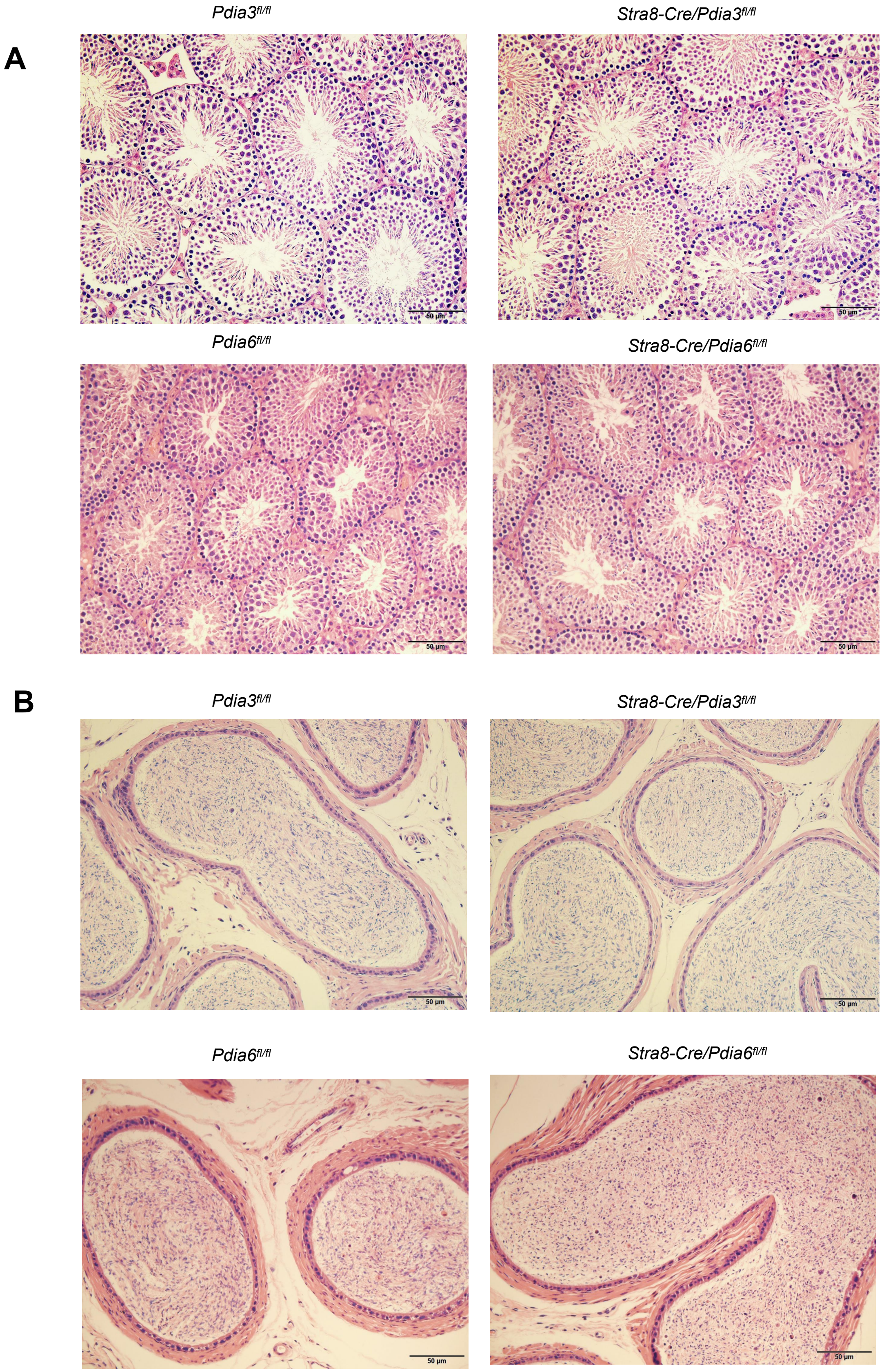

Supplemental Figure 3

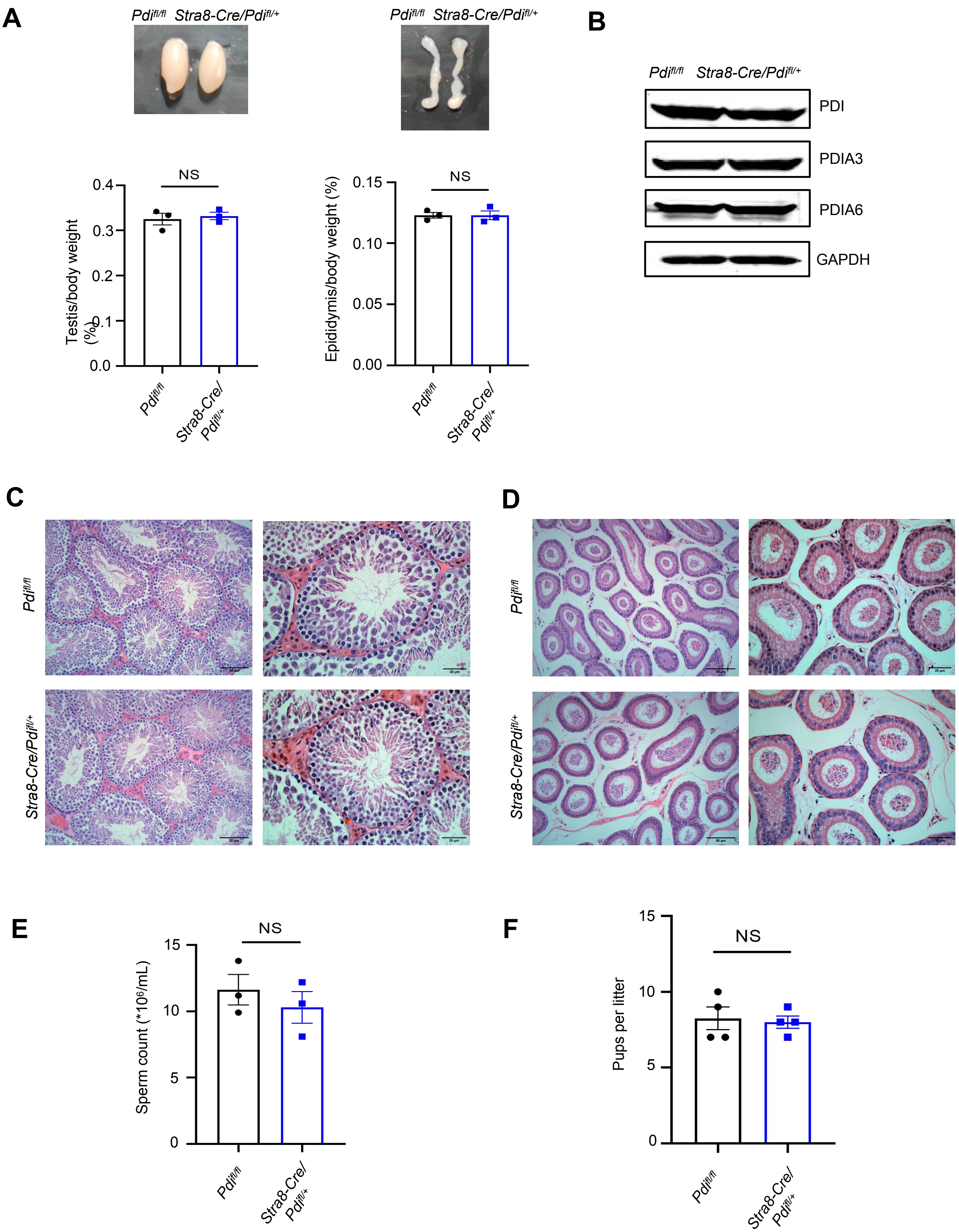

Supplemental Figure 4

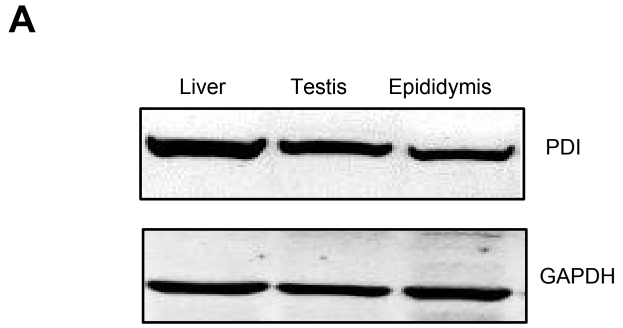

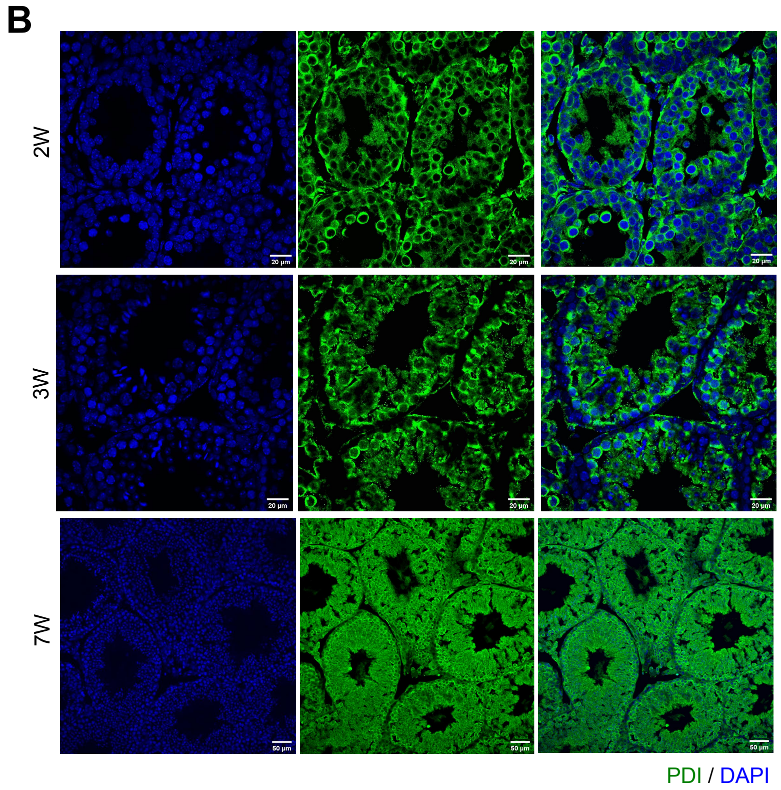

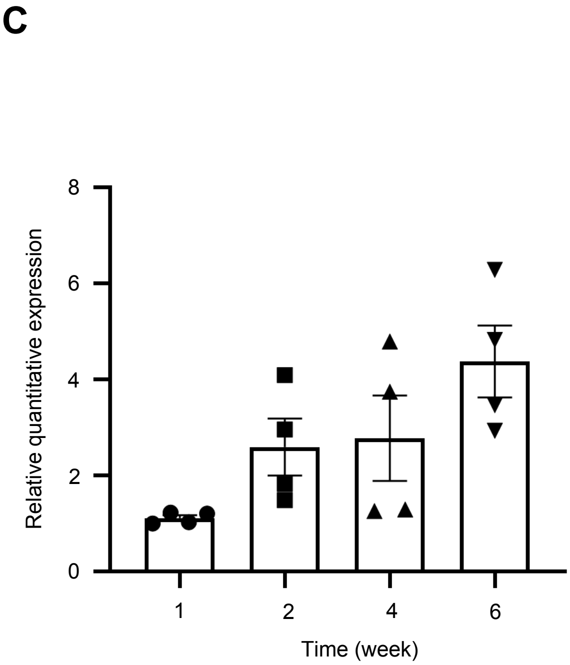

Supplemental Figure 5

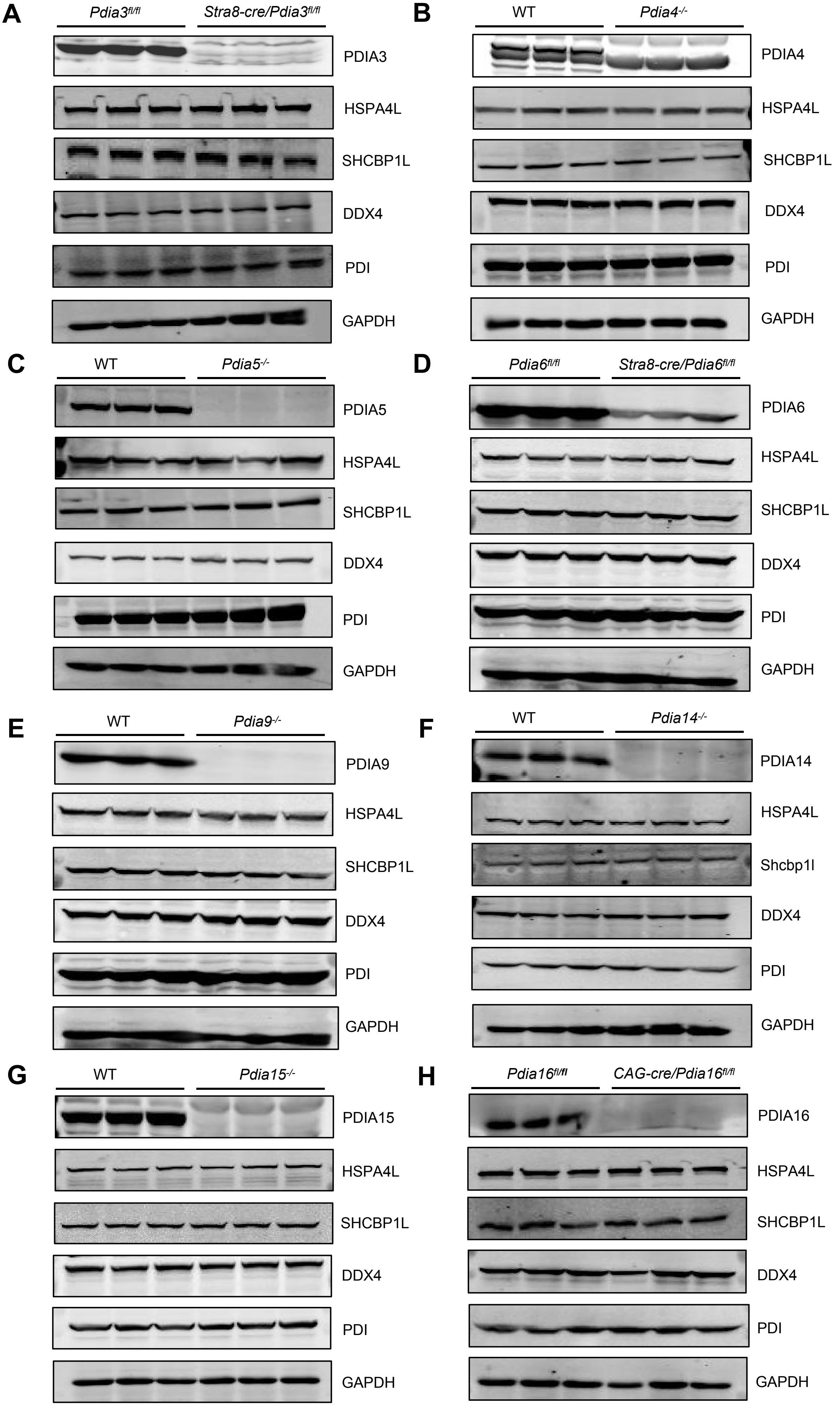

Supplemental figure legend and tables

Supplemental Figure 1. The absence of other PDI family members does not affect spermatogenesis. (A) H&E-stained testicular and epididymal sections of control, and *Pdia4*$^{-/-}$, *Pdia5*$^{-/-}$, *Pdia9*$^{-/-}$, *Pdia11*$^{-/-}$, *Pdia13*$^{-/-}$, *Pdia14*$^{-/-}$, *Pdia15*$^{-/-}$, *CAG-Cre/Pdia16*$^{fl/fl}$, and *Pdia19*$^{-/-}$ adult mice testes. Scale bars, 50 µm. **(B)** Western blotting analysis of PDIA2 and PDIA8 in testes and pancreas from adult mice. GAPDH as an internal loading control.

Supplemental Figure 2. Generation of *Stra8-Cre/Pdia3*$^{fl/fl}$ and *Stra8-Cre/Pdia6*$^{fl/fl}$ mice. (A) H&E-stained testes of control, *Stra8-Cre/Pdia3*$^{fl/fl}$, and *Stra8-Cre/Pdia6*$^{fl/fl}$ adult mice. *Stra8-Cre* mice were generated as previously described (Cell Research, 2017;27(10):1216-1230). Scale bars, 50 µm. **(B)** H&E-stained epididymis of control, *Stra8-Cre/Pdia3*$^{fl/fl}$, and *Stra8-Cre/Pdia6*$^{fl/fl}$ adult mice. Scale bars, 50 µm.

Supplemental Figure 3. *Stra8-Cre/Pdi*$^{fl/+}$ mice has normal fertility. (A) Images of testes and epididymis from control and *Stra8-Cre/Pdi*$^{fl/+}$ adult mice. Quantification of testis and epididymis weight to body-weight ratios (mean ± SEM, n=3, NS, not significant, Student's t test). **(B)** Western blotting analysis of PDIs protein in testes from control and *Stra8-Cre/Pdi*$^{fl/+}$ adult mice. GAPDH as an internal loading control. **(C)** H&E-stained testicular sections of control and *Stra8-Cre/Pdi*$^{fl/+}$ adult mice. Scale bars, 50 µm. **(D)** H&E-stained epididymal sections of control and *Stra8-Cre/Pdi*$^{fl/+}$ adult mice. Scale bars, 50 µm. **(E)** Sperm counts from control and *Stra8-Cre/Pdi*$^{fl/+}$ adult mice (mean ± SEM, n=3, NS, not significant, Student's t test). **(F)** Fertility test of control and *Stra8-Cre/Pdi*$^{fl/+}$ adult male mice (mean ± SEM, n=4, NS, not significant, Student's t test).

Supplemental Figure 4. PDI protein is widely expressed in testes. (A)

Western blotting analysis of PDI protein levels in adult mouse tissues, normalized to GAPDH. **(B)** Immunostaining of PDI in testes of mice at 2, 3, and 7 weeks. Scale bar, 20 μm. **(C)** qPCR analysis of *Pdi* mRNA levels at the indicated time points after birth in testes, normalized to GAPDH (mean ± SEM, n=4, Student's t test).

Supplemental Figure 5. The deletion of other PDI family members does not affect the expression of HSPA4L, SHCBP1L, and DDX4. (A-H) Western blotting analysis of the expression of HSPA4L, SHCBP1L, and DDX4 in testes of mice lacking *Pdia3, Pdia4, Pdia5, Pdia6, Pdia9, Pdia14, Pdia15 and Pdia16*. **A.** $Pdia3^{fl/fl}$ and $Stra8\text{-}Cre/Pdia3^{fl/fl}$. **B.** control and $Pdia4^{-/-}$. **C.** control and $Pdia5^{-/-}$. **D.** $Pdia6^{fl/fl}$ and $Stra8\text{-}Cre/Pdia6^{fl/fl}$. **E.** control and $Pdia9^{-/-}$. **F.** control and $Pdia14^{-/-}$. **G.** control and $Pdia15^{-/-}$. **H.** $Pdia16^{fl/fl}$ and $CAG\text{-}Cre/Pdia16^{fl/fl}$. GAPDH as an internal loading control.

Supplemental Table 1. Primer sequences used for genotyping

Genes	Forward	Reverse
PDI flox 1/2	TCAGCTTCTTGTTGGCACTAAGCTG	CTCCTCAATCTGTTCGCATTCCC
Stra8-cre 1/2	ACTCCAAGCACTGGGCAGAA	GCCACCATAGCAGCATCAAA
Stra8-cre 1/3	ACTCCAAGCACTGGGCAGAA	CGTTTACGTCGCCGTCCAG
UBC-cre 1/2	GCGGTCTGGCAGTAAAAACTATC	ACCATTGCCCCTGTTTCACT

Supplemental Table 2. Primer sequences used for quantitative PCR

Genes	Forward	Reverse
PDI	CTCGACAAAGATGGGGTTGT	GCAAGAACAGCAGGATGTGA
PDIA2	AGAATGGAAACCGCACAAAC	GACACCTTCCTCATCCTCCA
PDIA3	TGGTGTGGCCACTGTAAGAA	TTTAATTCACGGCCACCTTC
PDIA4	ATCGCCAAGATGGATGCTAC	CTTGGTCCTGCTCCTCTTTG
PDIA5	GGGAAGAACAGCAGACAAGC	TCTTACAGTGTGGGCACCAA

PDIA6	GGTGAGCTGCACCTTCTTTC	GCTGCTTTCTTCCATTCTGG
PDIA7	AAATGCAGGGTGCTGTTACC	AAGCCCTTCATGGTGTGTTC
PDIA8	TCTGCCTCTTTCGACTGGTT	TGCGCATGCTCTCTTCATAC
PDIA9	CCTGAAGATCATGGGGAAGA	TGGTCACAGCTCCTCCTTCT
PDIA10	TGTGCCTTCCTTTCTGCTTT	CGGACAAGAGGGACACATTT
PDIA11	CTGGTGTCCTGCTTGTCAGA	TAGTCCTTGGGCCCACATAG
PDIA12	TGTGCCTGCTTCTGATTTTG	CACTCCCTTCGAGAAACAGC
PDIA13	GGAATGAAGTTGGGCTTGAA	CCCAGACACTCTGTGAGCAA
PDIA14	GTGACAGCAAAACCAGAGCA	CCCGGTCTCCACAATTTCTA
PDIA15	AAAGGAATTCCCAGGCTTGT	CCGTTGTGTTCTCCCACTTT
PDIA16	GGAGAGGCCACTGACAGAAG	GTTAATCCCCAAGTGGCTCA
PDIA17	ACCGGCTCTACGCTTATGAA	CTGCCTTCTGGTCTCCTGAC
PDIA18	TTGTCACCTGATGGGCAGTA	TACTGCCCATCAGGTGACAA
PDIA19	GCTGGGGTCTTGGATTTTT	CGGCTCTCACTTTTCCTTTG
