# Supplementary material for: Protein disulfide isomerase is essential for spermatogenesis in mice
Source: JCI Insight. 2024 Jun 24;9(12):e177743. doi: 10.1172/jci.insight.177743 (PMC11383184; doi:10.1172/jci.insight.177743)
Supplement: Unedited blot and gel images [file jciinsight-9-177743-s261.pdf]

Fig1.b

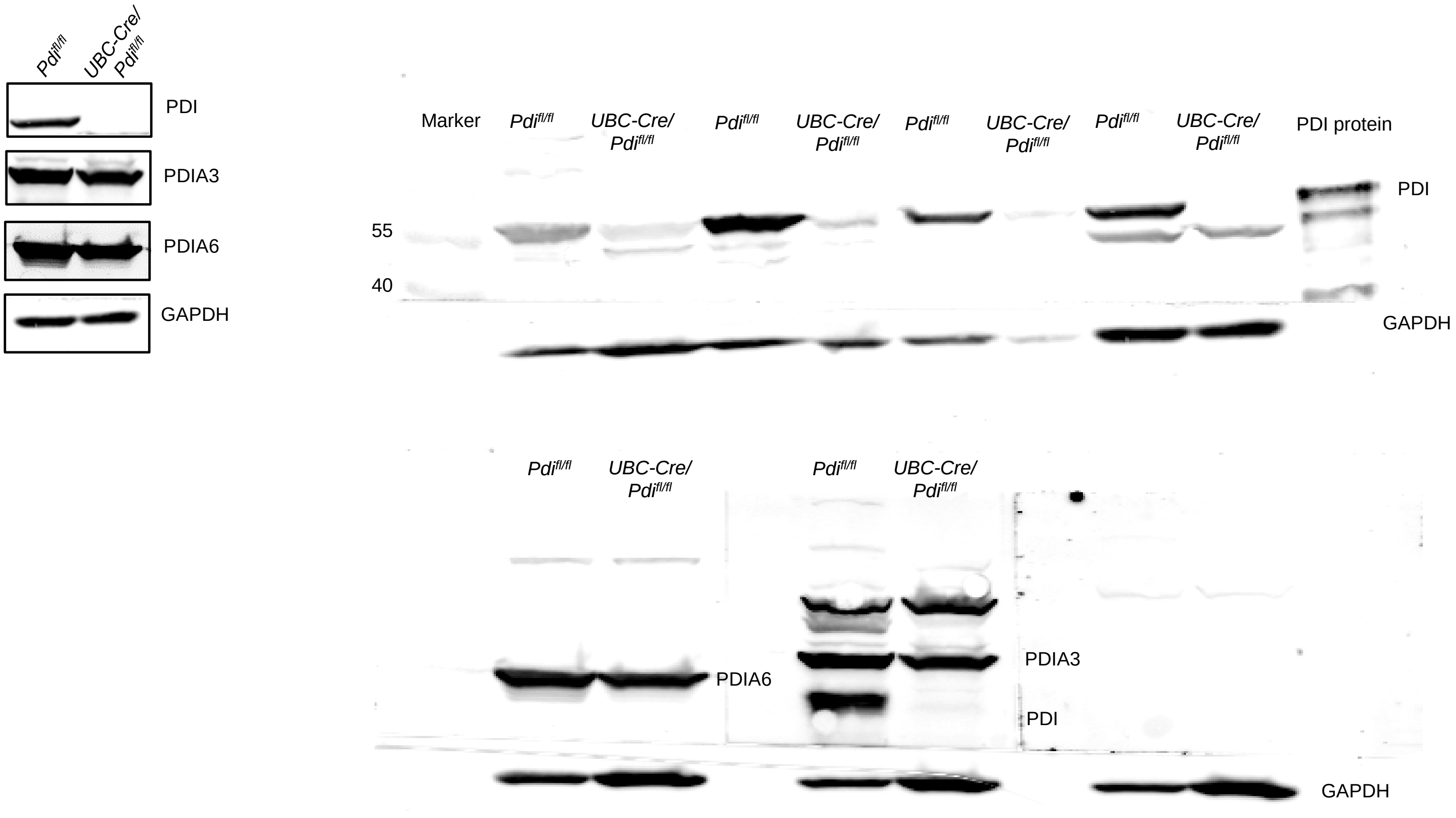

Antibody:
PDI (ABclonal, A19239)
PDIA3 (ABclonal, A1085)
PDIA6 (Abcam, ab154820)
GAPDH (Proteintech, 60004-1-Ig)

Fig2.d

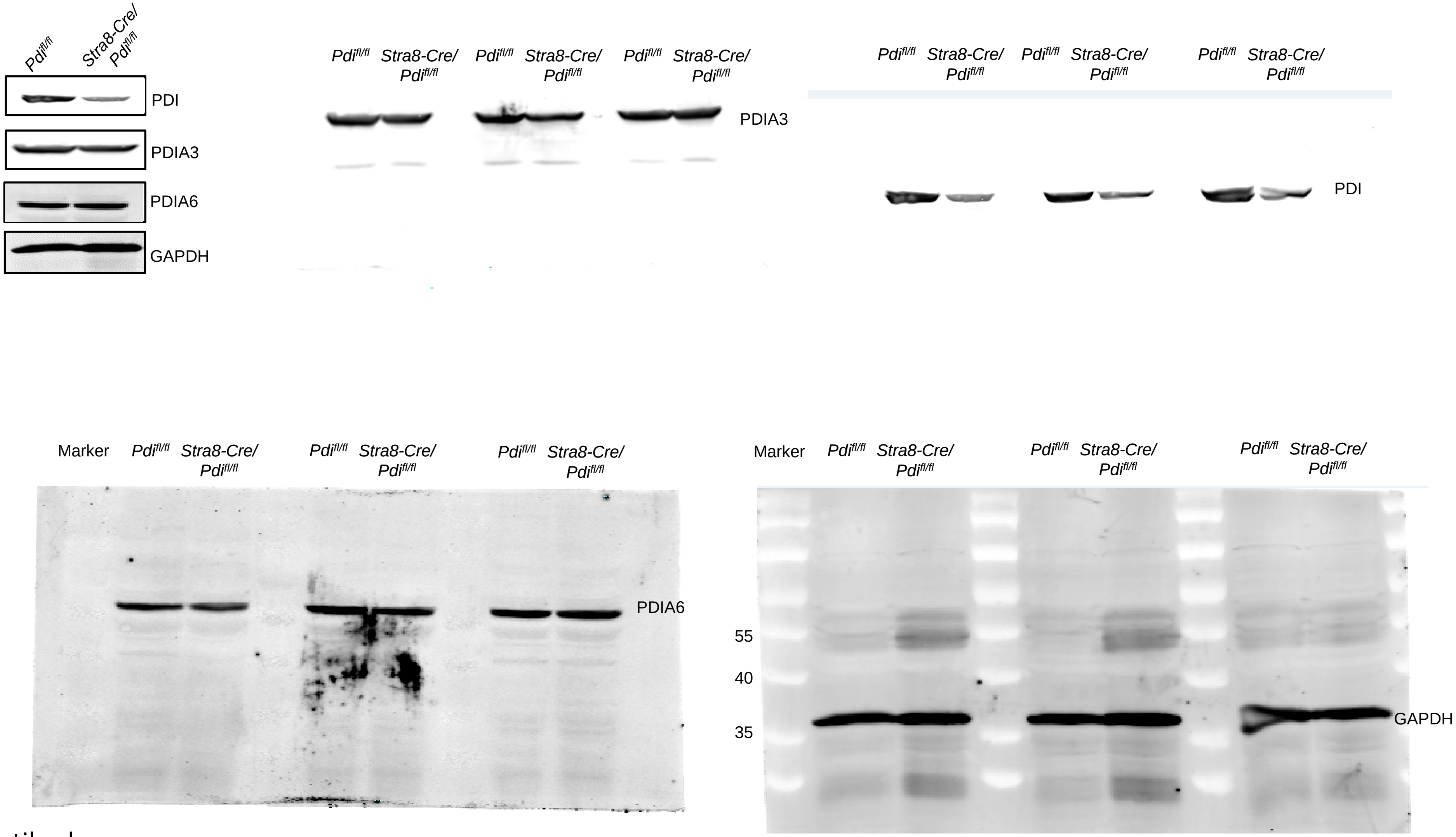

Antibody:

PDI (ABclonal, A19239)

PDIA3 (ABclonal, A1085)

PDIA6 (Abcam, ab154820)

GAPDH (Proteintech, 60004-1-Ig)

Fig5.a

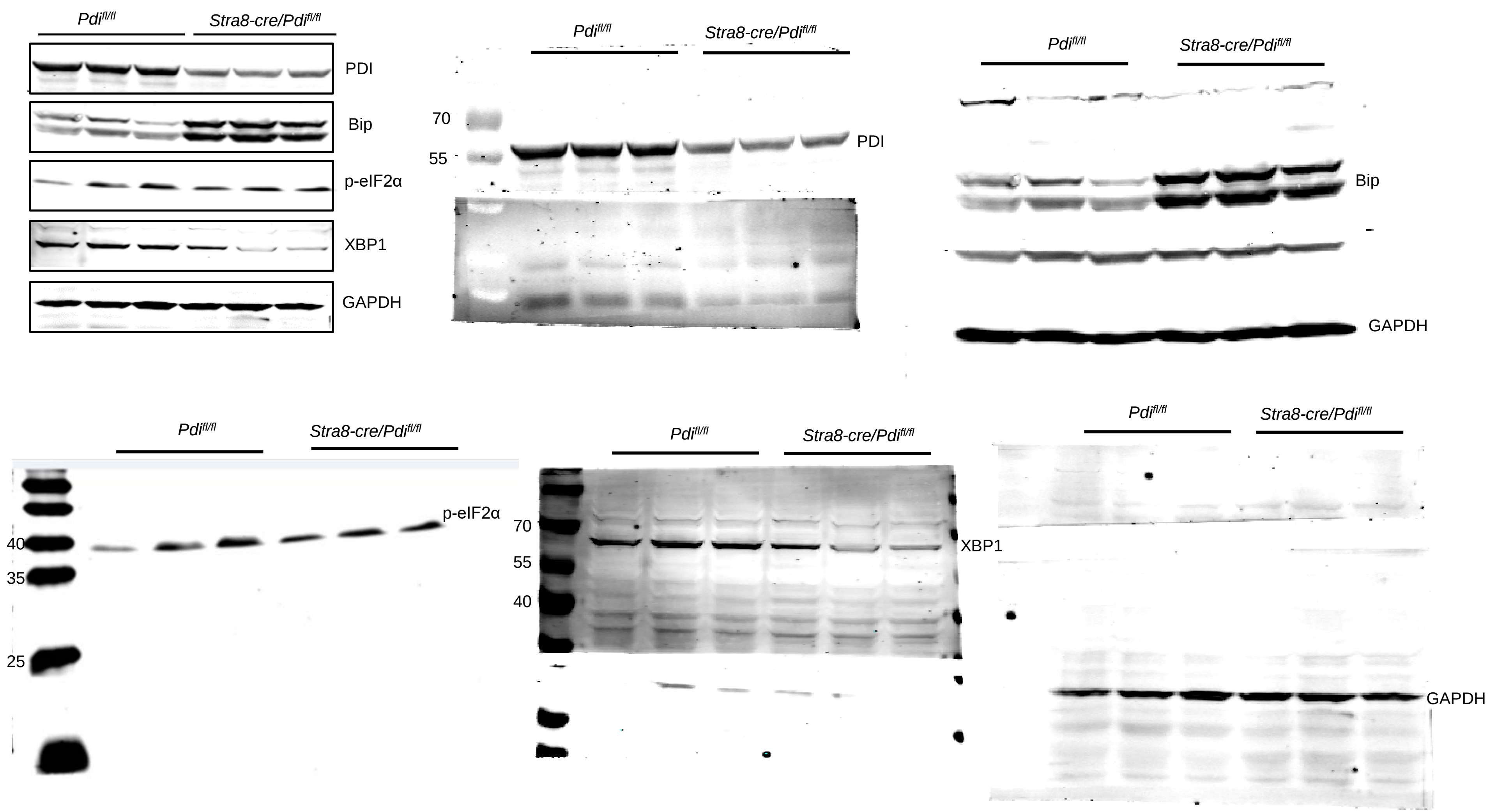

Antibody:
PDI (ABclonal, A19239)
GAPDH (Proteintech, 60004-1-Ig)
Bip (Cell Signaling Technology, C50B12)
p-eIF2α (Thermo Scientific, RD226932)
XBP1 (Cell Signaling Technology, 40435)

Fig5.b

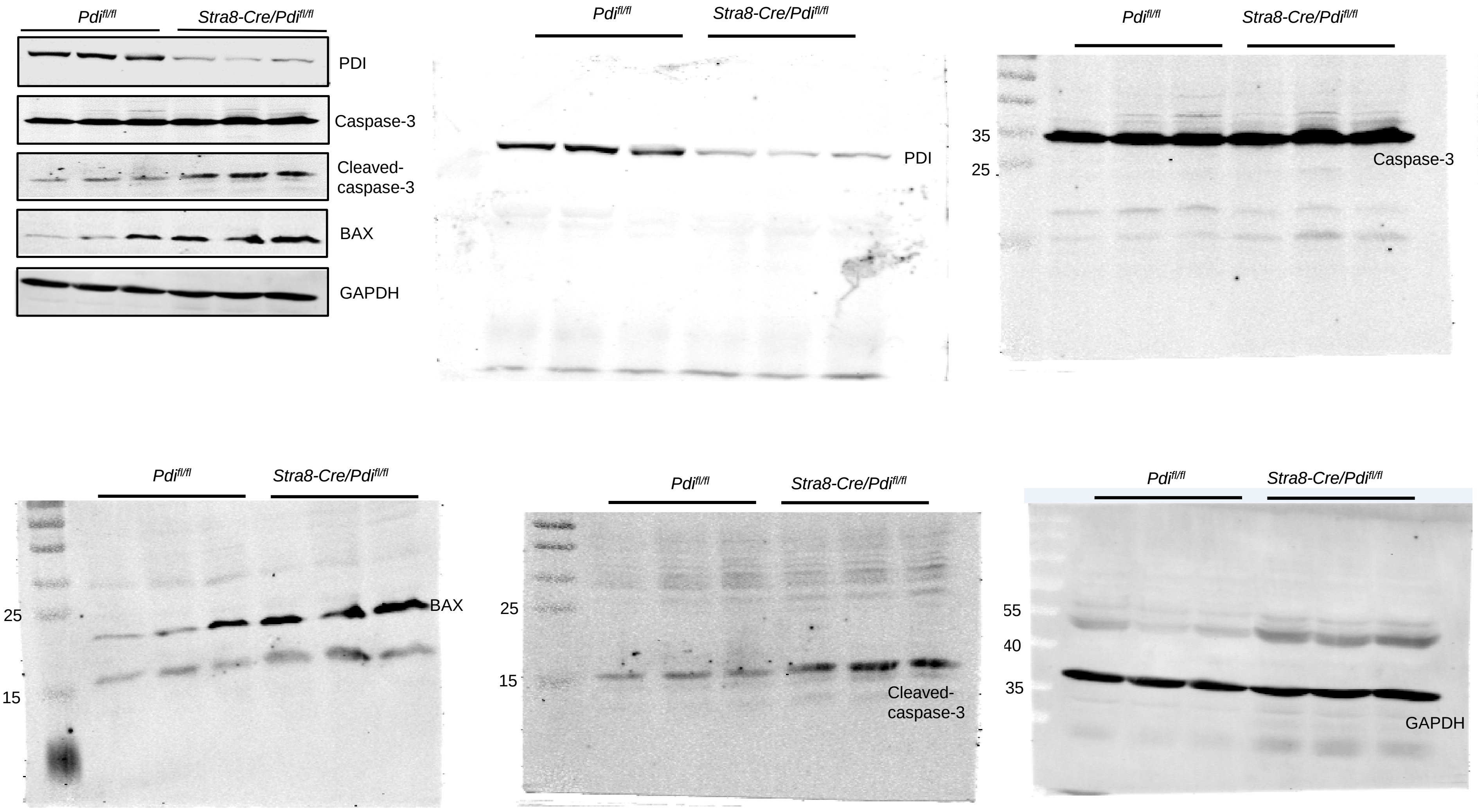

PDI (ABclonal, A19239)
GAPDH (Proteintech, 60004-1-Ig)
BAX (Abmart, T40051)
Caspase-3 (Abmart, TA6311)
cleaved Caspase-3 (Abmart, T61532)

Fig7.a

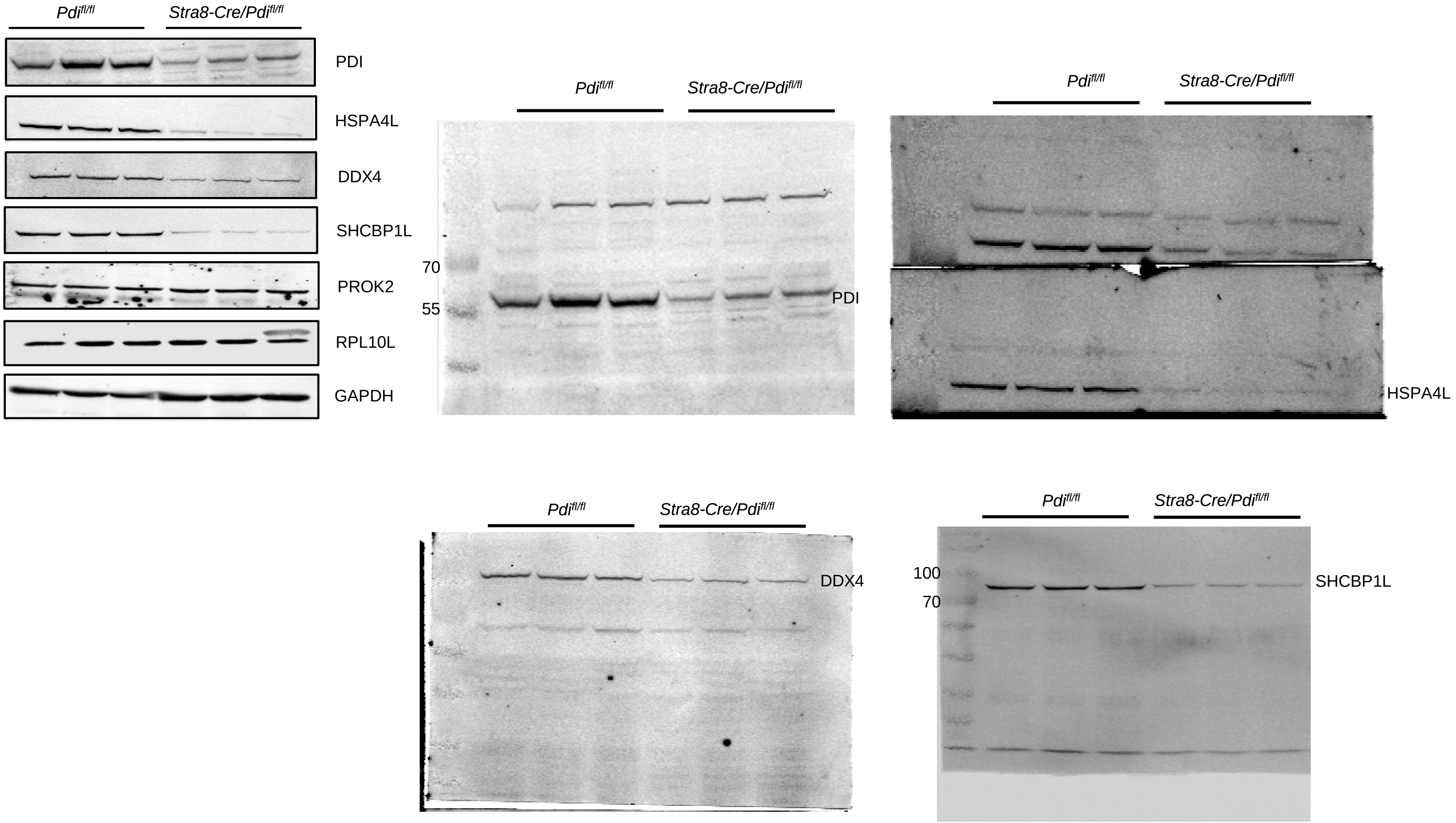

Antibody:
PDI (ABclonal, A19239)
HSPA4L (ABclonal, A17637)
SHCBP1L (Proteintech, 27108-1-AP)
DDX4 (Proteintech, 51042-1-AP)

Fig7.a

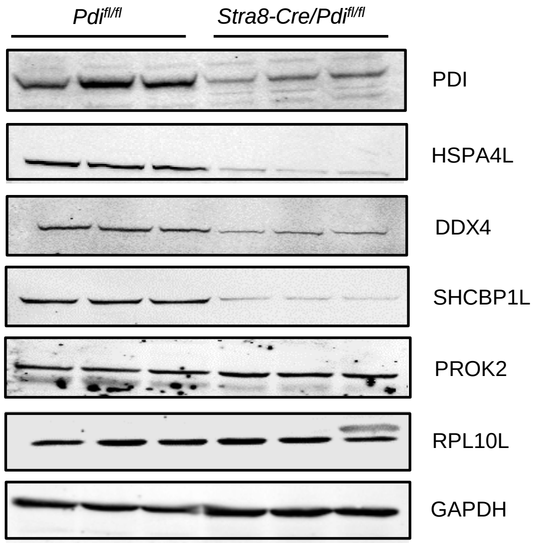

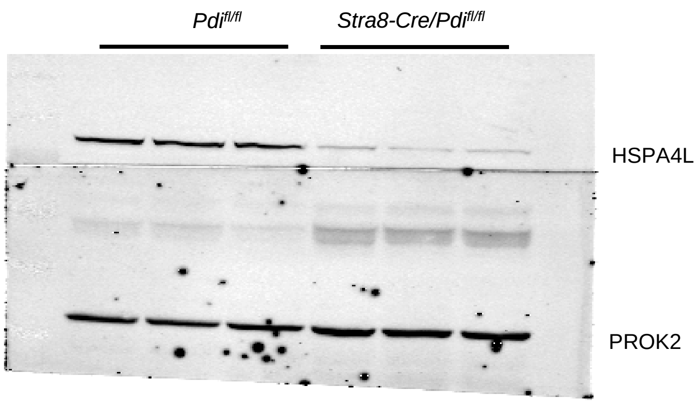

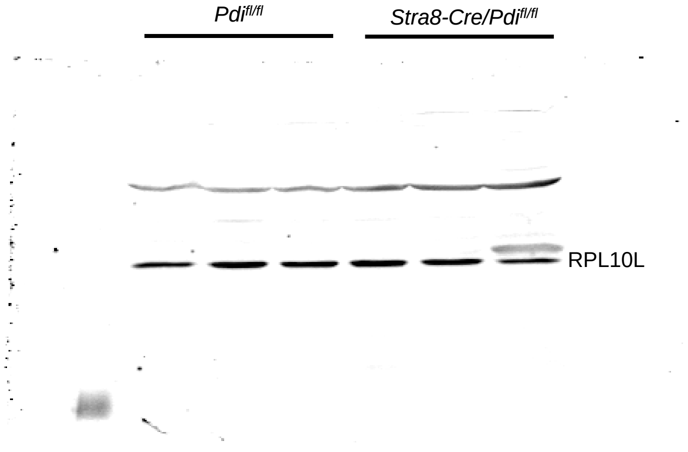

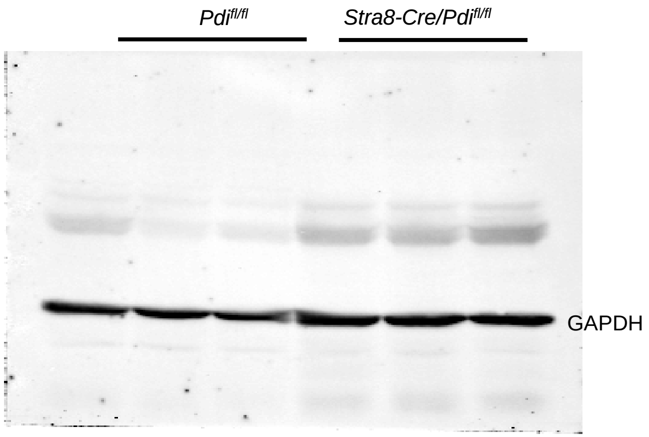

Antibody:
PROK2 (ABclonal, A6705)
RPL10L (Erpantech, AB-07-1234)
GAPDH (Proteintech, 60004-1-Ig)

Fig7.c

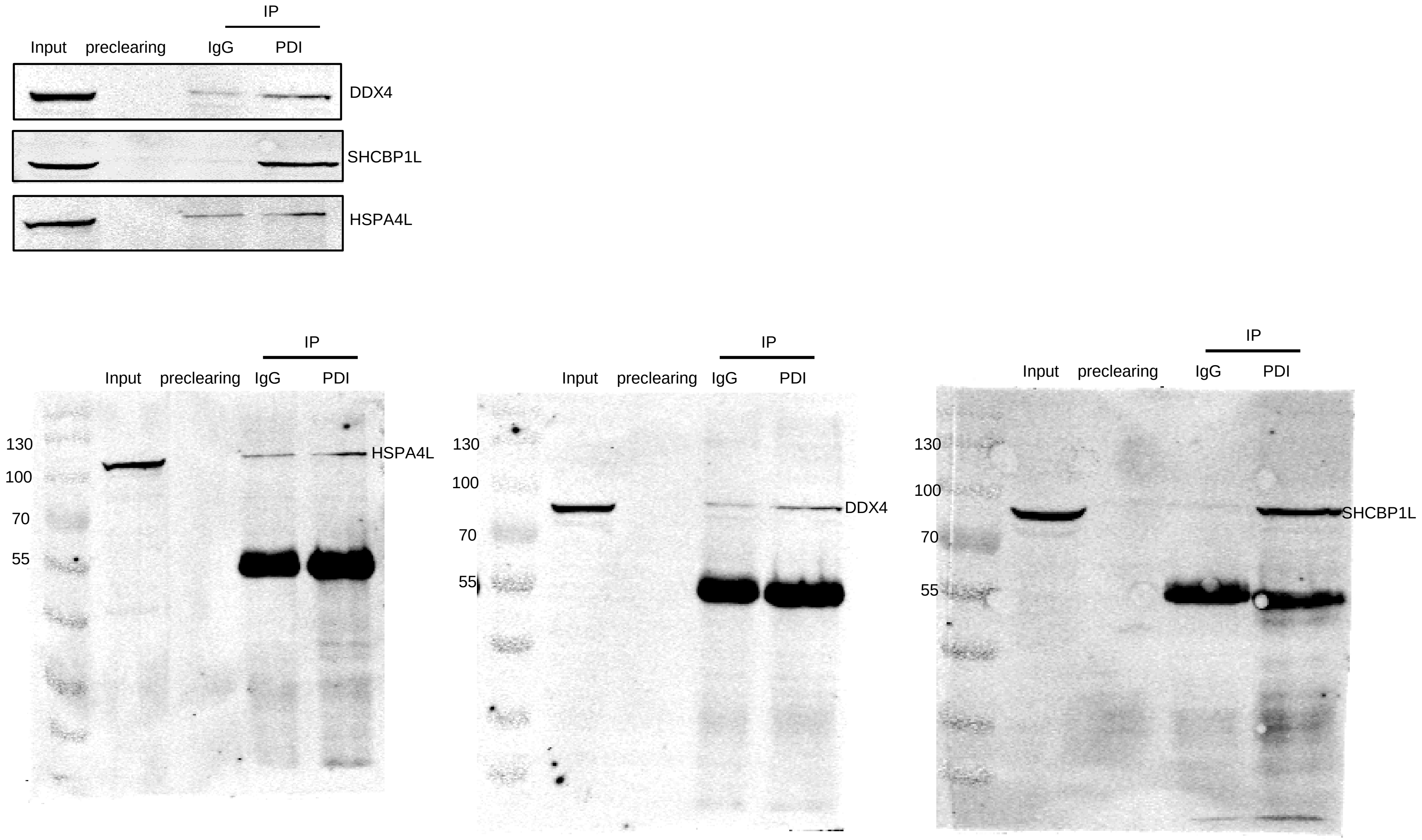

Antibody:
PDI (ABclonal, A19239)
HSPA4L (ABclonal, A17637)
SHCBP1L (Proteintech, 27108-1-AP)
DDX4 (Proteintech, 51042-1-AP)

Fig7.d

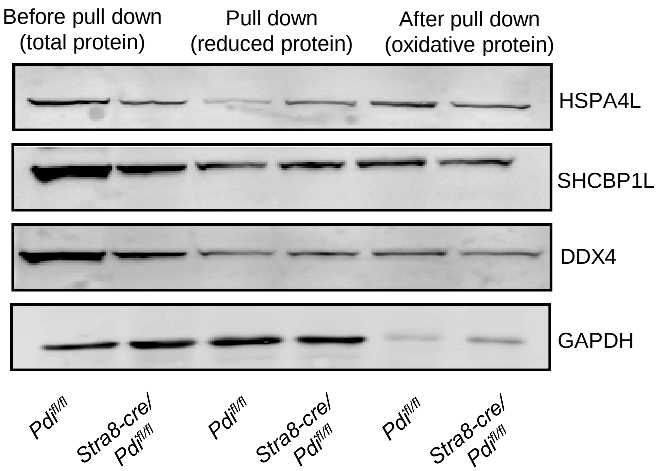

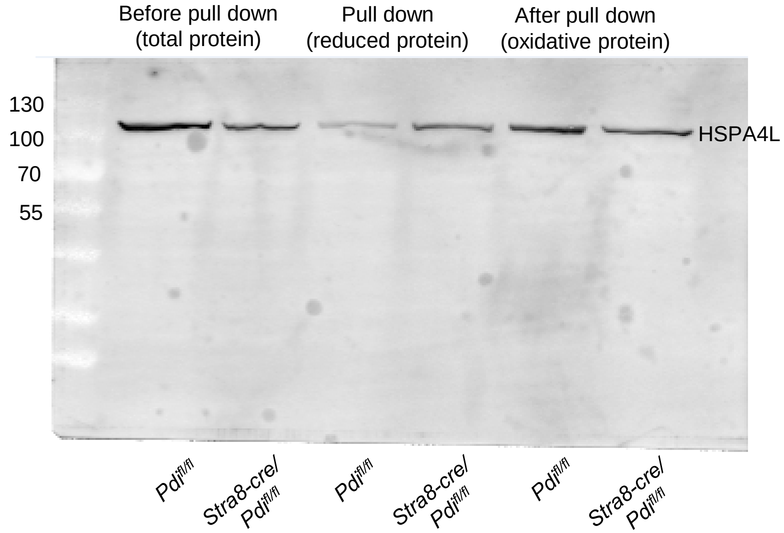

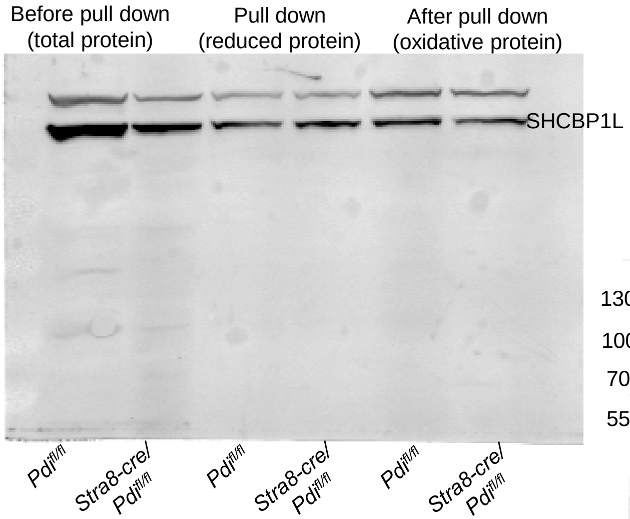

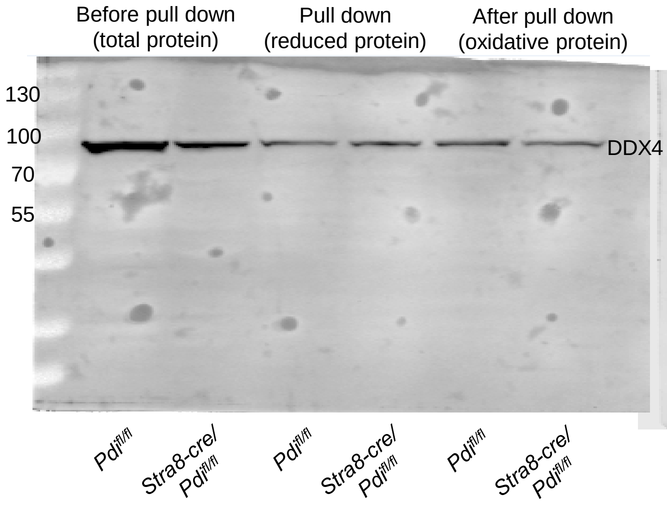

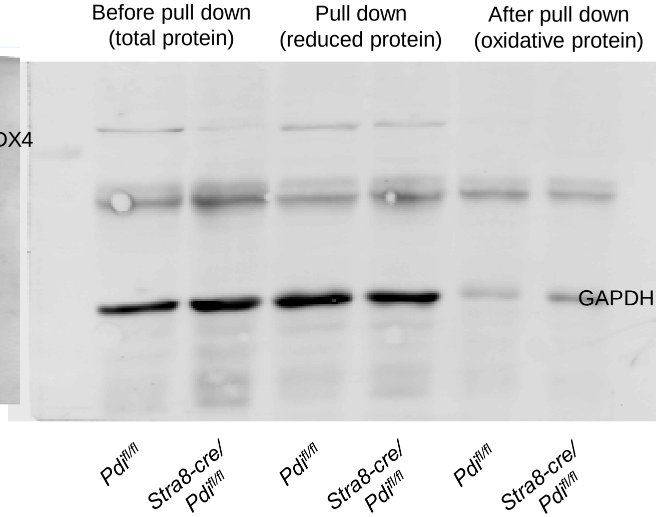

Antibody:
GAPDH (Proteintech, 60004-1-Ig)
HSPA4L (ABclonal, A17637)
SHCBP1L (Proteintech, 27108-1-AP)
DDX4 (Proteintech, 51042-1-AP)

FigS1. b

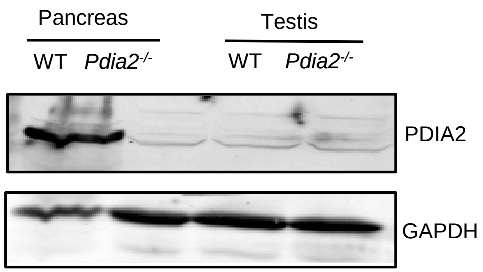

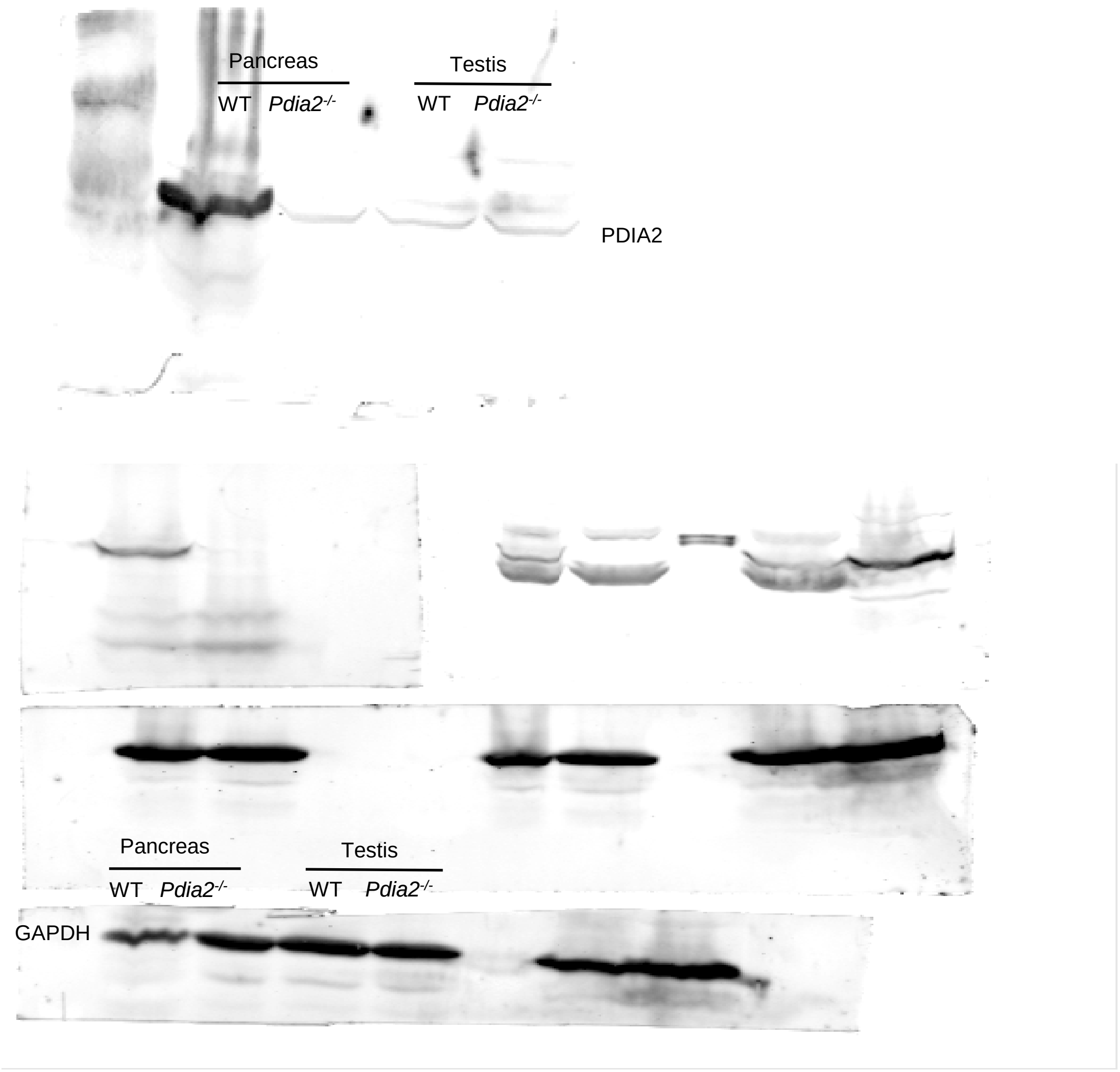

Antibody:
PDIA2 (Invitrogen, PA5-112644)
GAPDH (Proteintech, 60004-1-Ig)

FigS1.b

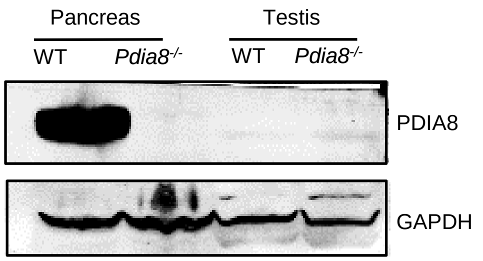

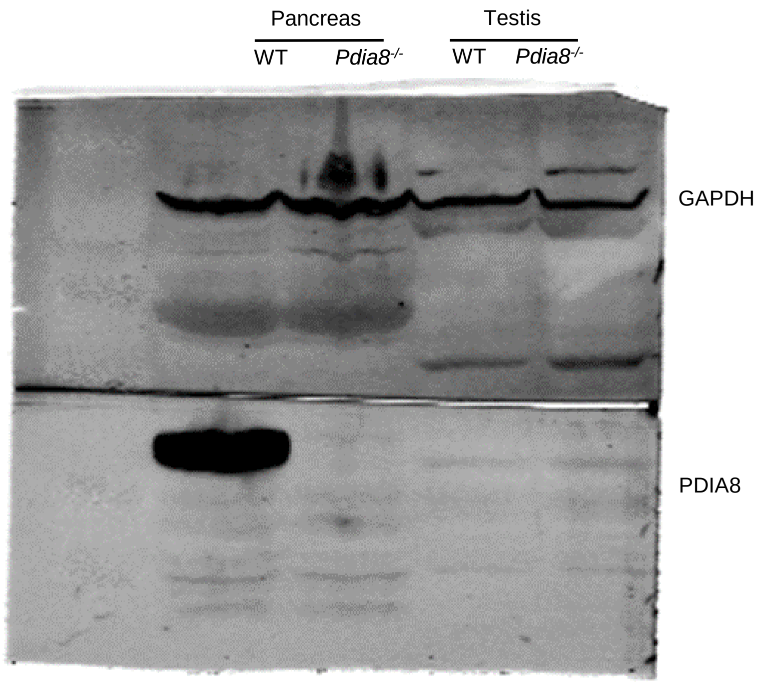

Antibody:
PDIA8 (Abcam, ab181172)
GAPDH (Proteintech, 60004-1-Ig)

FigS3.b

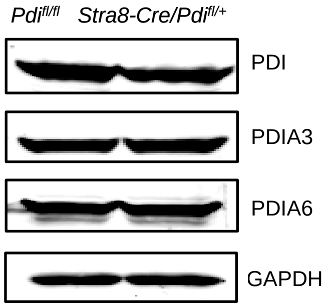

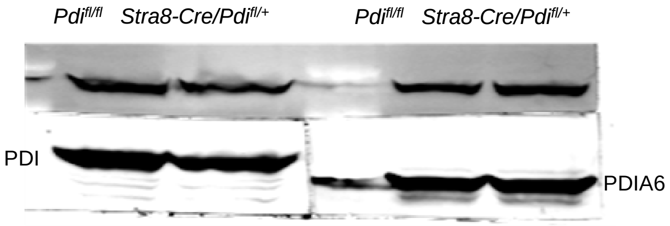

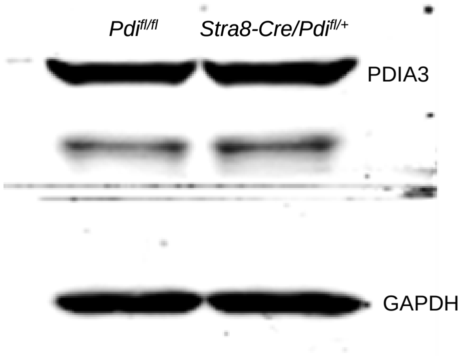

Antibody:
PDI (ABclonal, A19239)
PDIA3 (ABclonal, A1085)
PDIA6 (Abcam, ab154820)
GAPDH (Proteintech, 60004-1-Ig)

FigS4.a

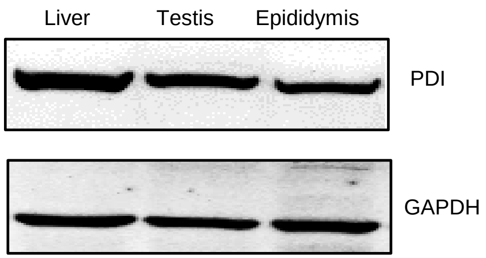

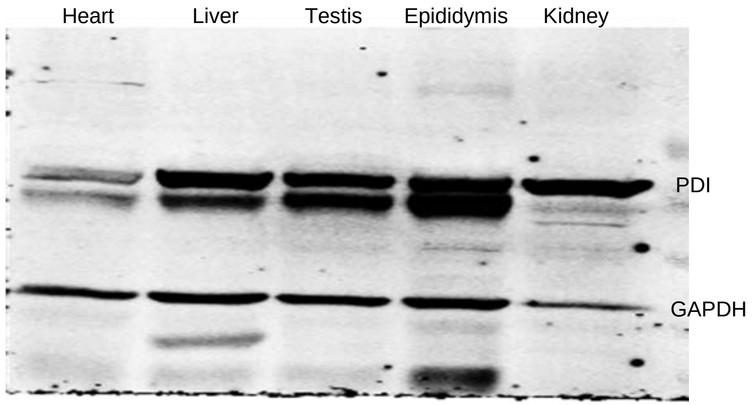

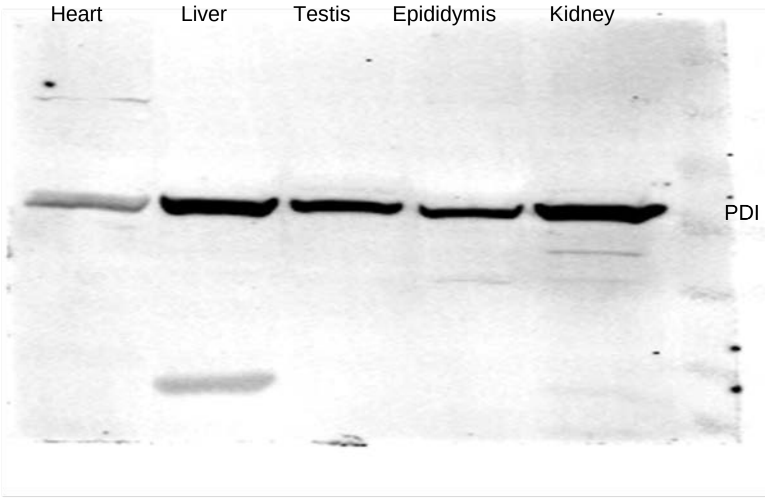

Antibody:
PDI (ABclonal, A19239)
GAPDH (Proteintech, 60004-1-Ig)

Fig. S5a

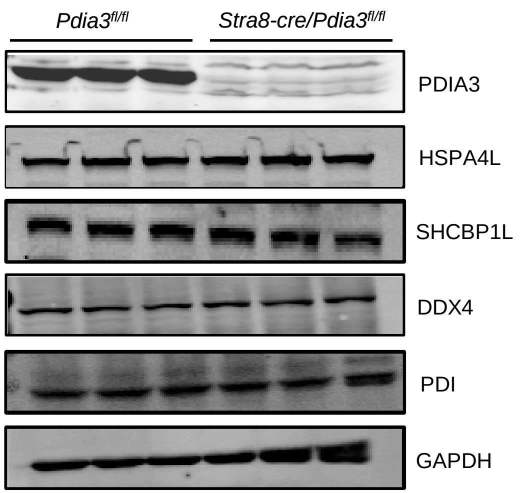

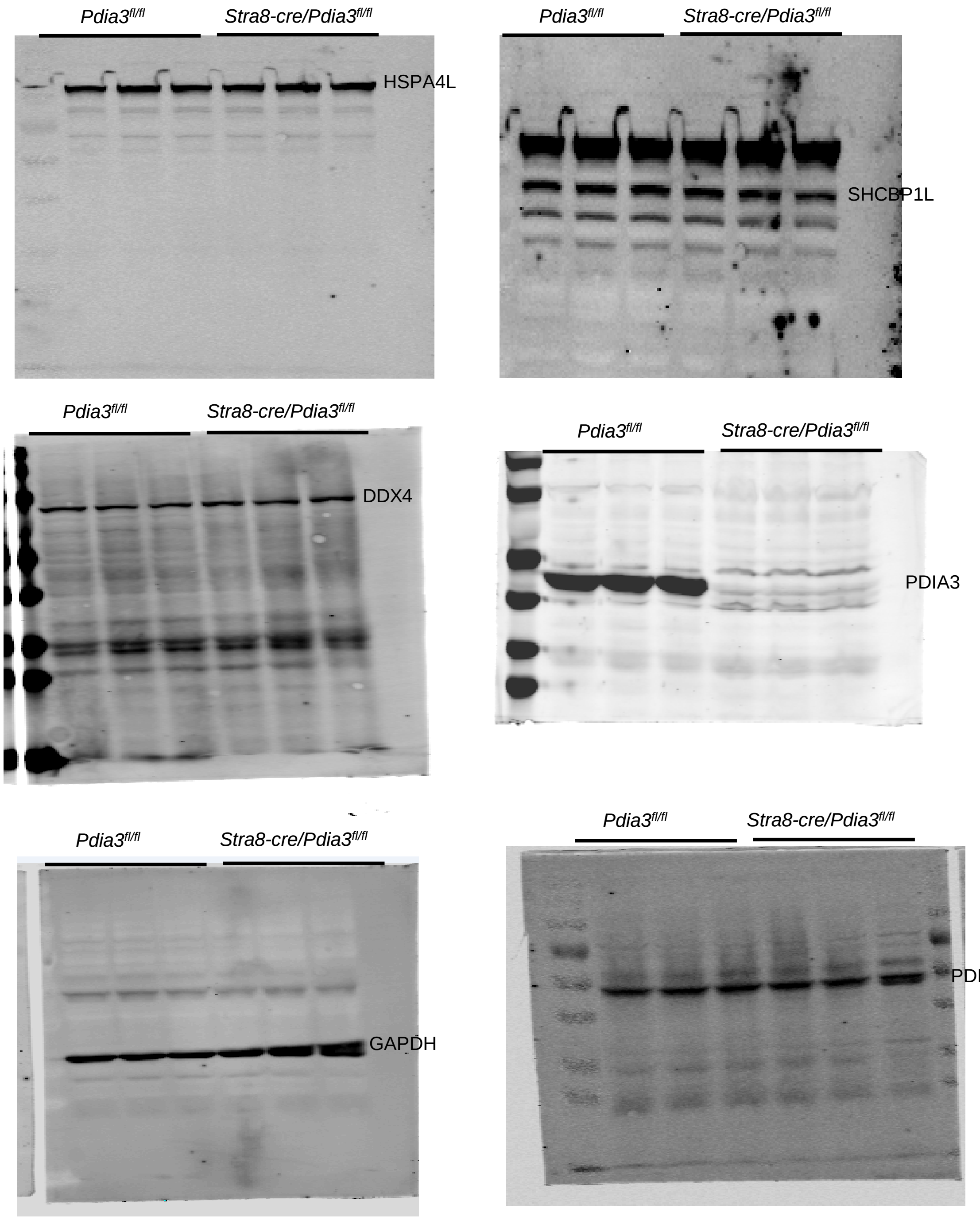

Antibody:
PDI (ABclonal, A19239)
HSPA4L (ABclonal, A17637)
SHCBP1L(Proteintech, 27108-1-AP)
DDX4(Proteintech, 51042-1-AP)
GAPDH (Proteintech, 60004-1-Ig)
PDIA3 (ABclonal, A1085)

Fig. S5b

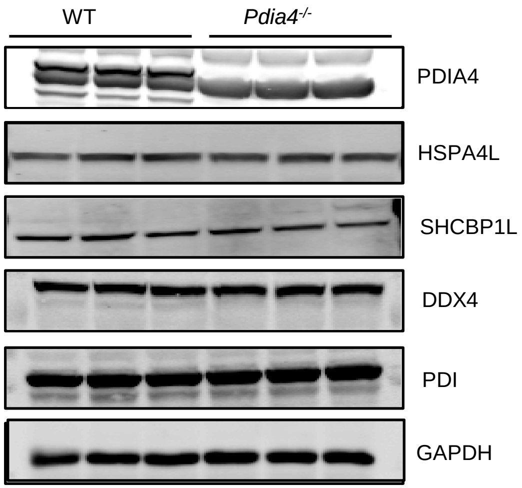

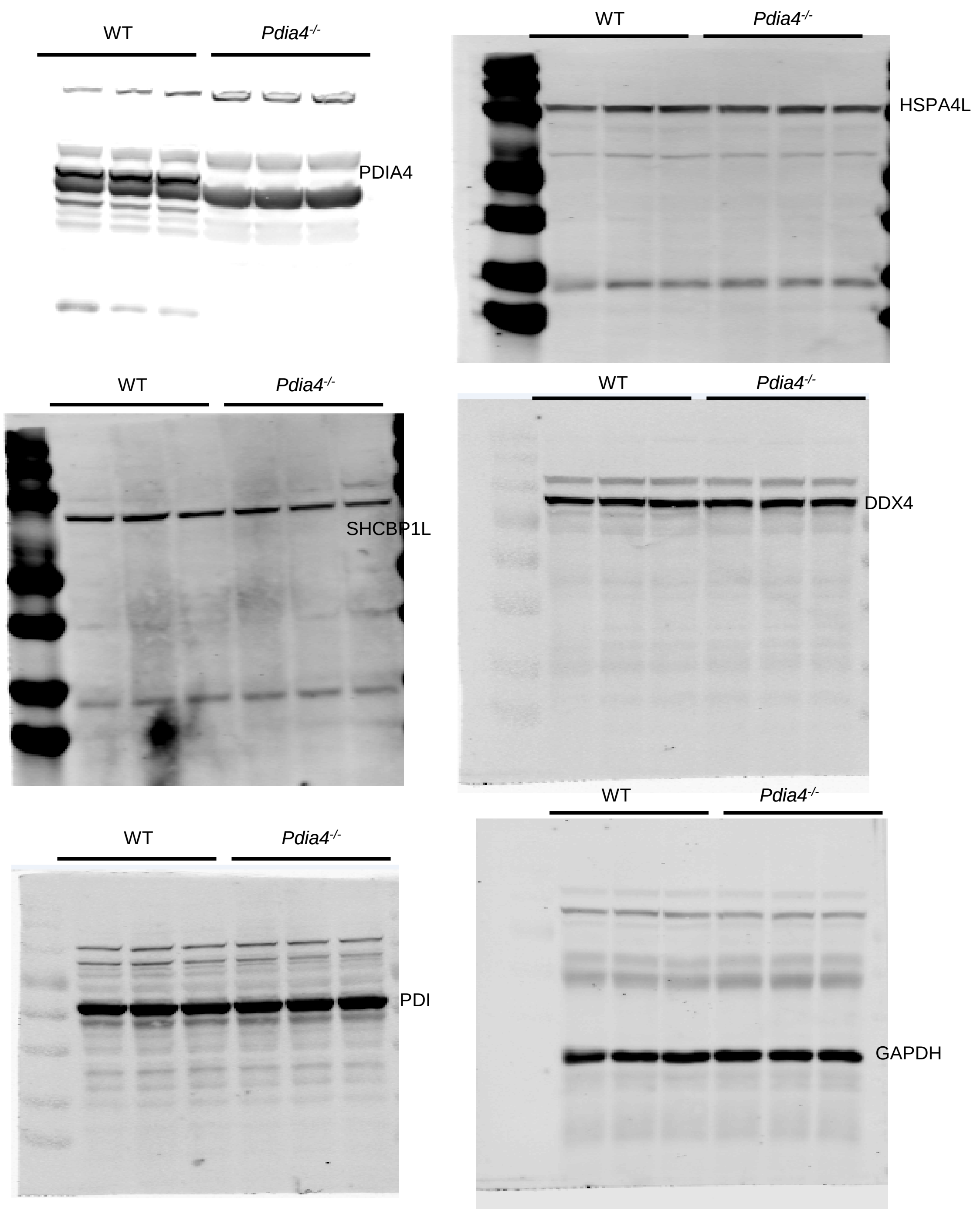

Antibody:
PDI (ABclonal, A19239)
HSPA4L (ABclonal, A17637)
SHCBP1L(Proteintech, 27108-1-AP)
DDX4(Proteintech, 51042-1-AP)
GAPDH (Proteintech, 60004-1-Ig)
PDIA4 (Cell Signaling Technology, 5033)

Fig. S5c

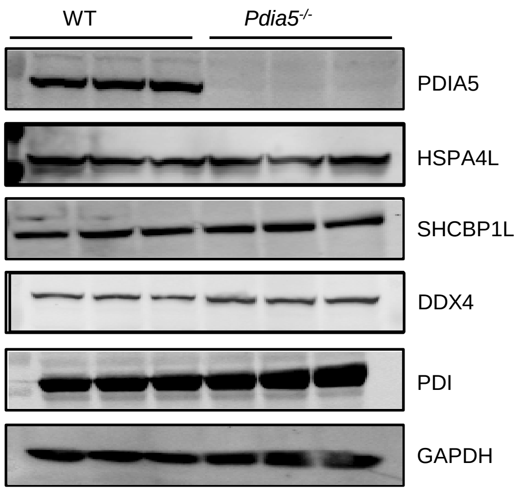

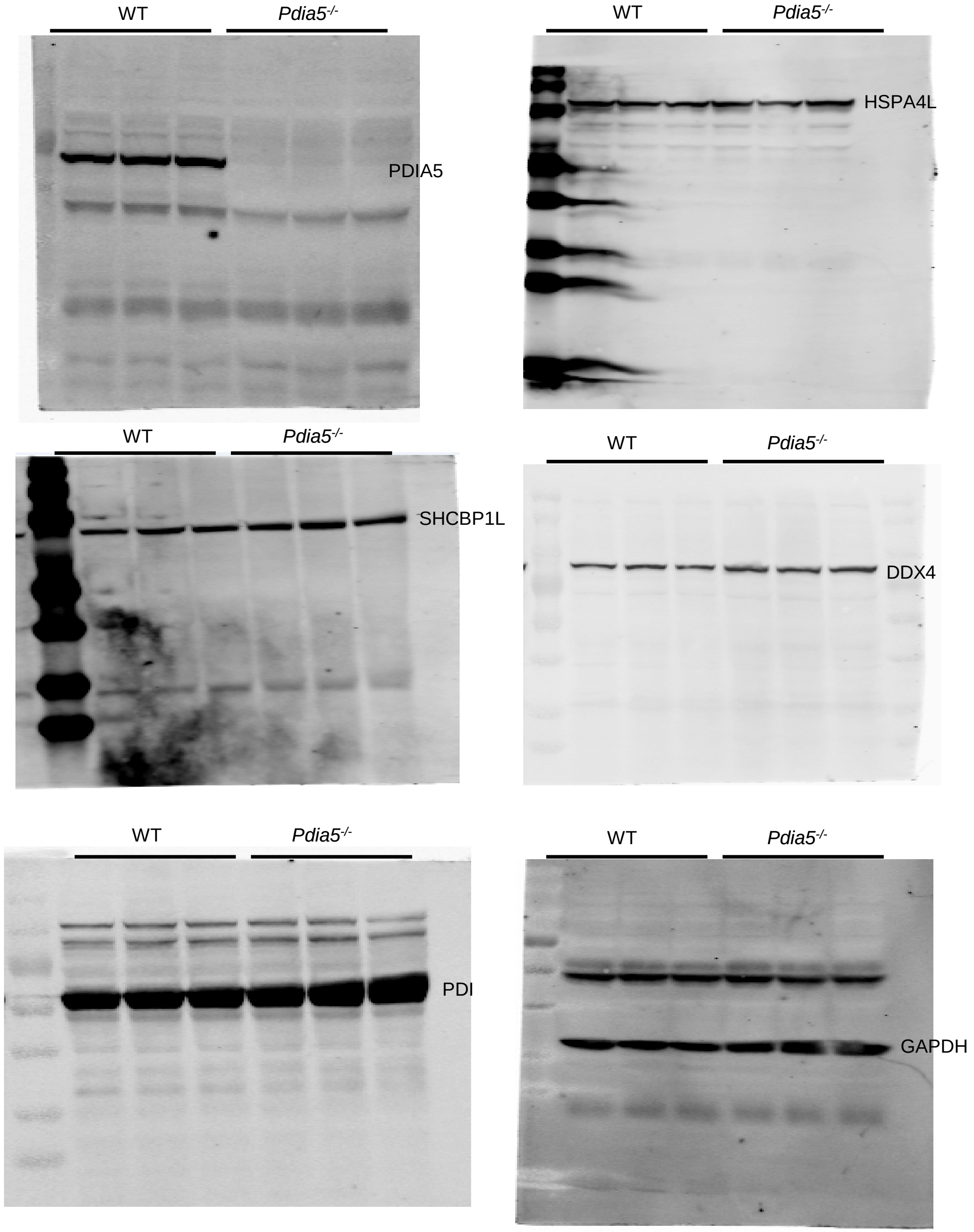

Antibody:
PDI (ABclonal, A19239)
HSPA4L (ABclonal, A17637)
SHCBP1L(Proteintech, 27108-1-AP)
DDX4(Proteintech, 51042-1-AP)
GAPDH (Proteintech, 60004-1-Ig)
PDIA5 (Proteintech, 15545-1-AP)

Fig. S5d

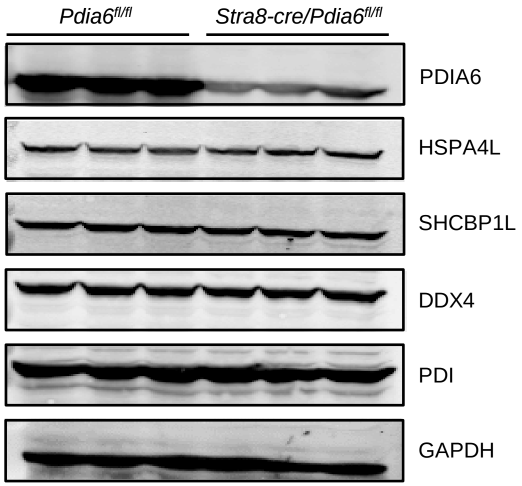

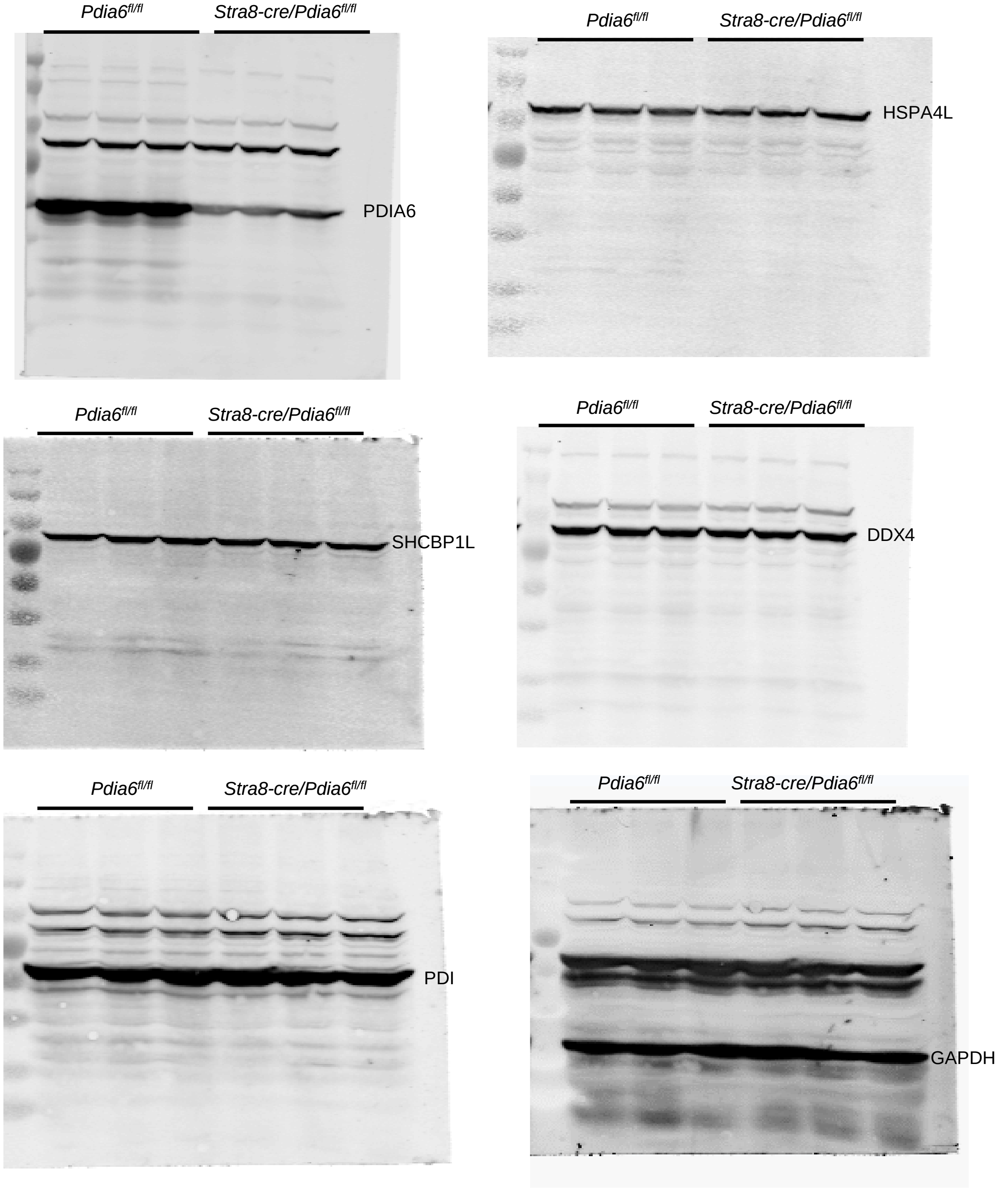

Antibody:
PDI (ABclonal, A19239)
HSPA4L (ABclonal, A17637)
SHCBP1L(Proteintech, 27108-1-AP)
DDX4(Proteintech, 51042-1-AP)
GAPDH (Proteintech, 60004-1-Ig)
PDIA6 (Abcam, ab154820)

Fig. S5e

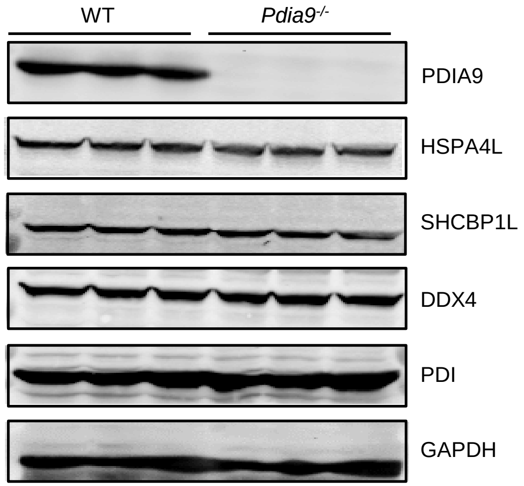

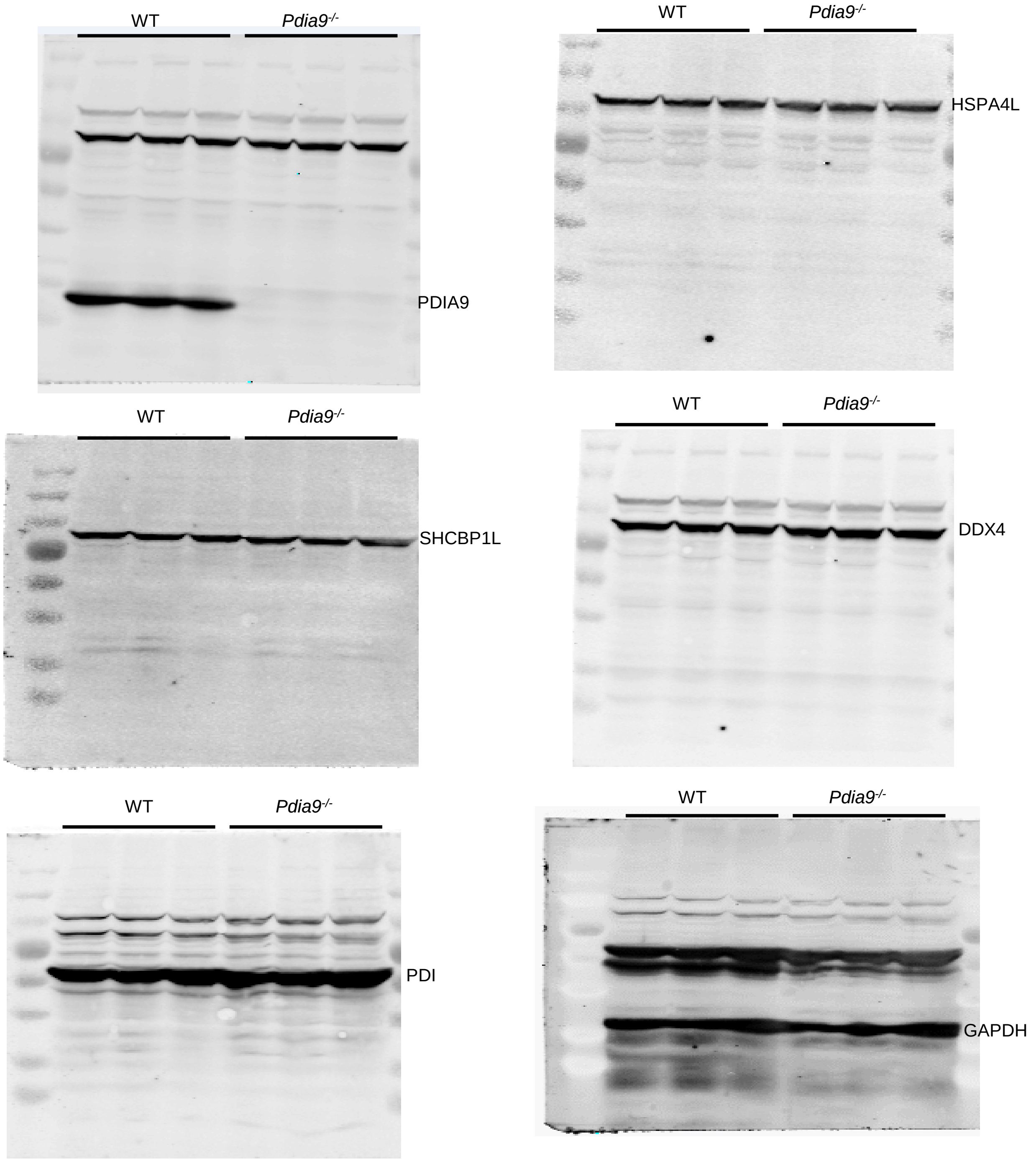

Antibody:
PDI (ABclonal, A19239)
HSPA4L (ABclonal, A17637)
SHCBP1L(Proteintech, 27108-1-AP)
DDX4(Proteintech, 51042-1-AP)
GAPDH (Proteintech, 60004-1-Ig)
PDIA9 (Abcam, ab11420)

Fig. S5f

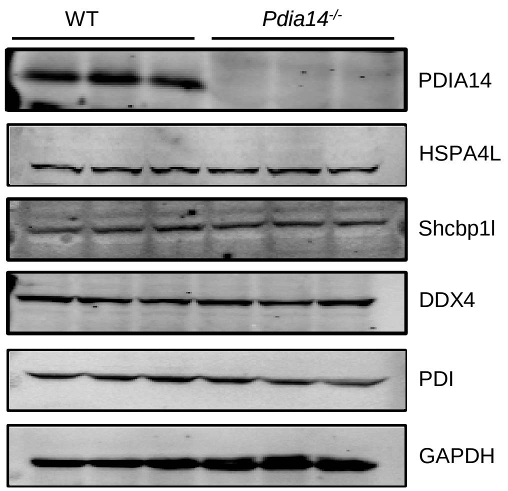

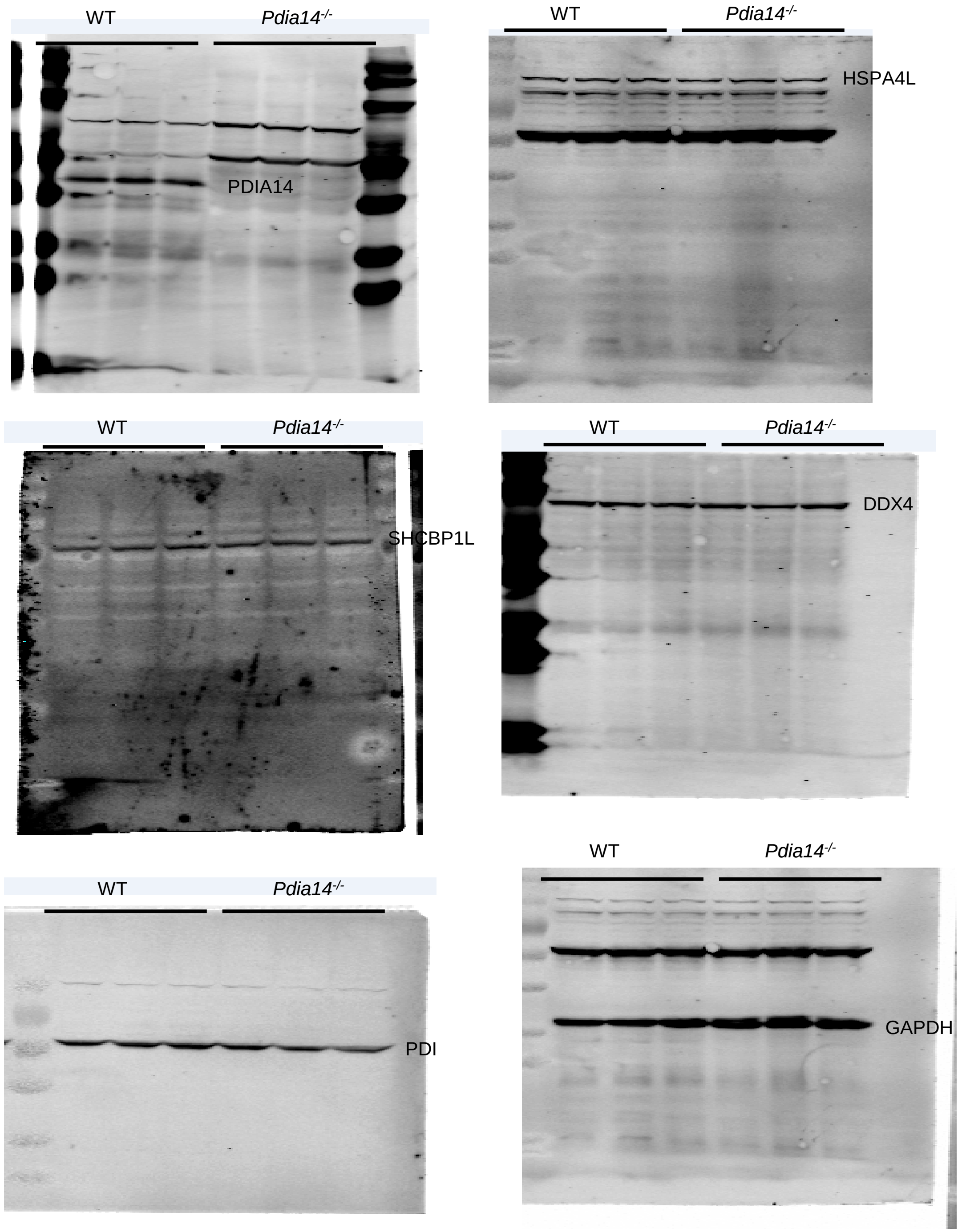

Antibody:
PDI (ABclonal, A19239)
HSPA4L (ABclonal, A17637)
SHCBP1L(Proteintech, 27108-1-AP)
DDX4(Proteintech, 51042-1-AP)
GAPDH (Proteintech, 60004-1-Ig)
PDIA14 (Proteintech, 21348-1-AP)

Fig. S5g

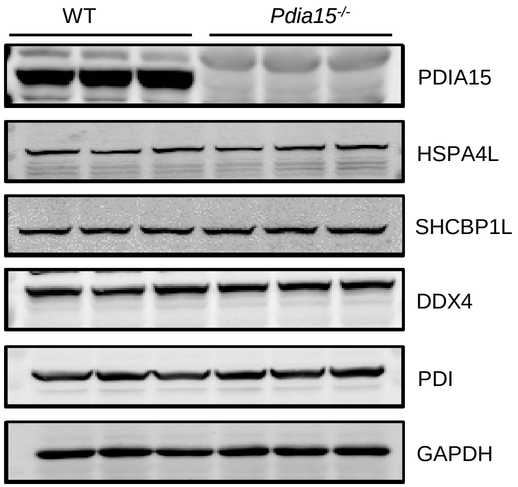

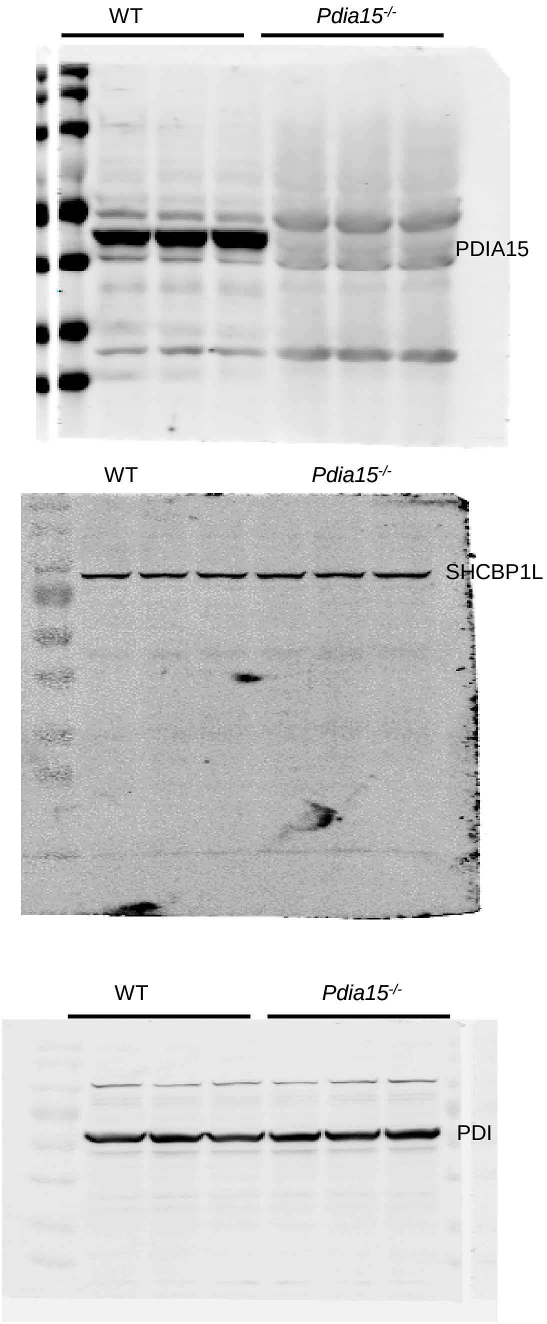

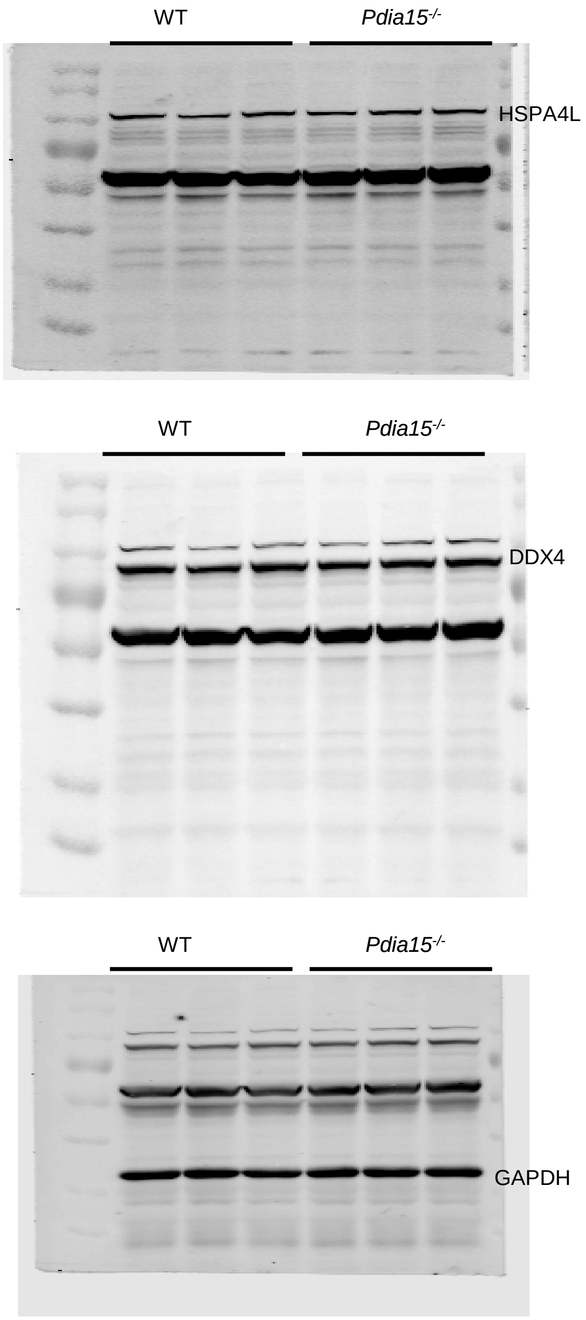

Antibody:
PDI (ABclonal, A19239)
HSPA4L (ABclonal, A17637)
SHCBP1L(Proteintech, 27108-1-AP)
DDX4(Proteintech, 51042-1-AP)
GAPDH (Proteintech, 60004-1-Ig)
PDIA15 (Proteintech, 19834-1-AP)

Fig. S5h

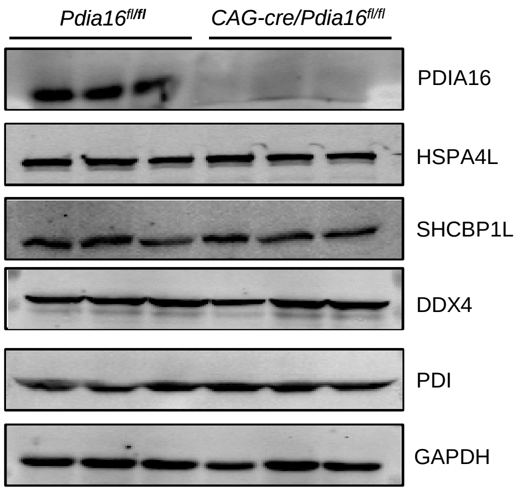

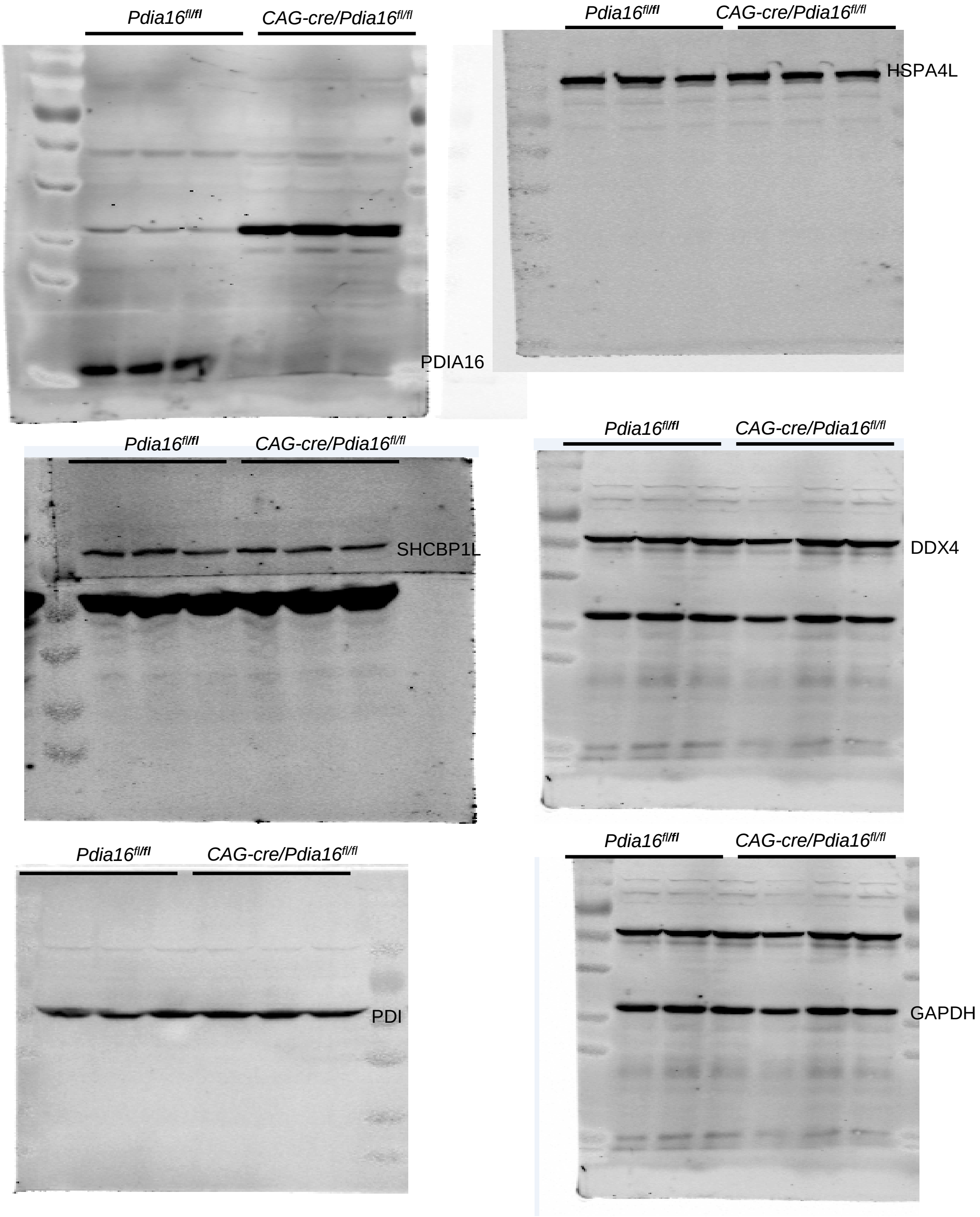

Antibody:

PDI (ABclonal, A19239)

HSPA4L (ABclonal, A17637)

SHCBP1L(Proteintech, 27108-1-AP)

DDX4(Proteintech, 51042-1-AP)

GAPDH (Proteintech, 60004-1-Ig)

PDIA16 (Novus Biologicals, SR1258)
